# Newly synthesized 3-(4-chloro-phenyl)-3-hydroxy-2,2-dimethyl-propionic acid methyl ester derivatives selectively inhibit the proliferation of colon cancer cells

**DOI:** 10.1039/c9ra10950a

**Published:** 2020-02-28

**Authors:** Samir M. El Rayes, Ahmed Aboelmagd, Mohamed S. Gomaa, Walid Fathalla, Ibrahim A. I. Ali, Faheem H. Pottoo, Firdos Alam Khan

**Affiliations:** Department of Chemistry, Faculty of Science, Suez Canal University Ismailia Egypt; Department of Pharmaceutical, College of Clinical Pharmacy, Imam Abdulrahman Bin Faisal University P. O. Box 1982 Dammam 31441 Kingdom of Saudi Arabia; Department of Physics and Math., Faculty of Engineering, Port-Said University Port-Said Egypt; Department of Pharmacology, College of Clinical Pharmacy, Imam Abdulrahman Bin Faisal University P. O. Box 1982 Dammam 31441 Kingdom of Saudi Arabia fhpottoo@iau.edu.sa; Department of Stem Cell Research, Institute of Research and Medical Consultations (IRMC), Imam Abdulrahman Bin Faisal University P. O. Box 1982 Dammam 31441 Saudi Arabia fakhan@iau.edu.sa

## Abstract

A series of 24 compounds were synthesized based on structure modification of the model methyl-3-(4-chlorophenyl)-3-hydroxy-2,2-dimethylpropanoate as potent HDACIs. Saponification and hydrazinolysis of the model ester afforded the corresponding acid and hydrazide, respectively. The model ester was transformed into the corresponding trichloroacetimidate or acetate by the reaction with trichloroacetonitrile and acetic anhydride, respectively. *N*-Alkyl-3-(4-chlorophenyl)-3-hydroxy-2,2-dimethylpropan-amides and methyl-2-[(3-(4-chlorophenyl)-3-hydroxy-2,2-dimethylpropanoyl)amino] alkanoates were obtained by the reaction of corresponding acid or hydrazide with amines and amino acid esters *via* DCC and azide coupling methods. Methyl-3-aryl-3-(4-chlorophenyl)-2,2-dimethylpropanoates were obtained in good yields and short reaction time from the corresponding trichloroacetimidate or acetate by the reaction with C-active nucleophiles in the presence of TMSOTf (0.1 eq.%) *via* C–C bond formation. The antiproliferative and apoptotic activity were further studied with molecular docking. The 48 post-treatments showed that out of 24 compounds, 12 compounds showed inhibitory actions on HCT-116 cells, we have calculated the inhibitory action (IC_50_) of these compounds on HCT-116 and we have found that the IC_50_ values were in between 0.12 mg mL^−1^ to 0.81 mg mL^−1^. The compounds (7a & 7g) showed highest inhibitory activity (0.12 mg mL^−1^), whereas compound 7d showed the lowest inhibitory activity (0.81 mg mL^−1^). We have also examined inhibitory action on normal and non-cancerous cells (HEK-293 cells) and confirmed that action of these compounds was specific to cancerous cells. The cancerous cells were also examined for nuclear disintegration through staining with DAPI, (4′,6-diamidino-2-phenylindole) is a blue-fluorescent DNA stain, and we have found that there was loss of DAPI staining in the compound treated cancerous cells. The compounds were found to potentially act through the HSP90 and TRAP1 mediated signaling pathway. Compounds 7a and 7g showed the highest selectivity to TRAP1 which explained its superior activity.

## Abstract 2

A series of 24 compounds were synthesized based on structure modification of the model methyl-3-(4-chlorophenyl)-3-hydroxy-2,2-dimethylpropanoate as potent HDACIs.

## Graphical abstract

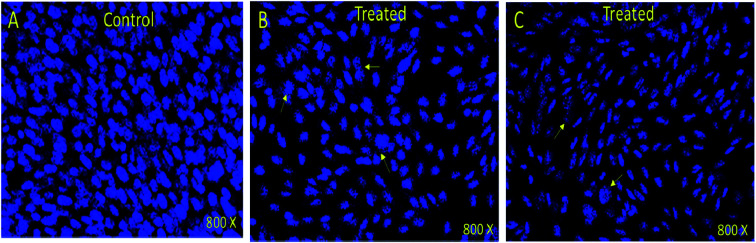

## Introduction

Malignancy is one of the significant factors behind loss of life in the developed countries.^1–3^ Chemotherapy with cytotoxic medications is one of the primary approaches to dealing with established malignancy.^4,5^ The primary drawbacks of malignancy chemotherapy is the severe poisonous results such as emesis and myelosuppression, as well as the insufficient selectivity of the drugs against cyst tumor cellular material in comparison with normal cellular material.^1,6^ Hence, searching for newer anticancer drugs is a never-ending job. Key interactions at protein–protein interfaces constitute important targets for small molecule inhibition because of their specific arrangements and biological importance.^7^

Recently, we reported the synthesis of 3-(4-(2-chloroacetamido)phenyl)-3-hydroxy-2,2-dimethylpropanoate and 3-(4-chlorophenyl)-3-hydroxy-2,2-dimethylpropanoate as potent HDACIs.^8^ We also showed that the antiproliferative activity for these compounds against HeLa cells (IC_50_; 11–0.69 μM) was excellent in comparison with the standard drug doxorubicin (IC_50_; 2.29 μM).^8^ Our compounds are peptide in nature that mimic peptide inhibitors and non-peptide inhibitors, together with being more suitable for pharmaceutical manipulations and development and this work provides integration of our research effort in discovering and modifying new anticancer agents.^8,9^

## Results and discussions

### Chemistry

**Scheme 1:**
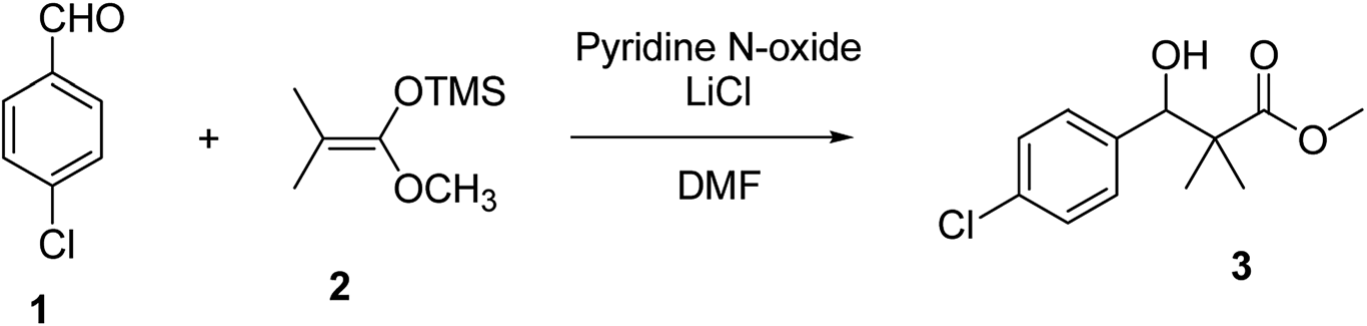
Synthesis of methyl-3-hydroxy-2,2-dimethyl-3-(4-chlorophenyl)propanoate (3).

Herein, we report the synthesis of a series of compounds based on structure modification of the model methyl-3-(4-chlorophenyl)-3-hydroxy-2,2-dimethylpropanoate (3) as promising HDACIs. The model compound 3 was prepared by the reaction of 4-chlorobenzaldehyde 1 and trimethylsilyl ketene acetal 2 in the presence of pyridine-*N*-oxide and LiCl in DMF at room temperature under nitrogen atmosphere to afford 3 in 69% yield, Scheme 1.

**Scheme 2:**
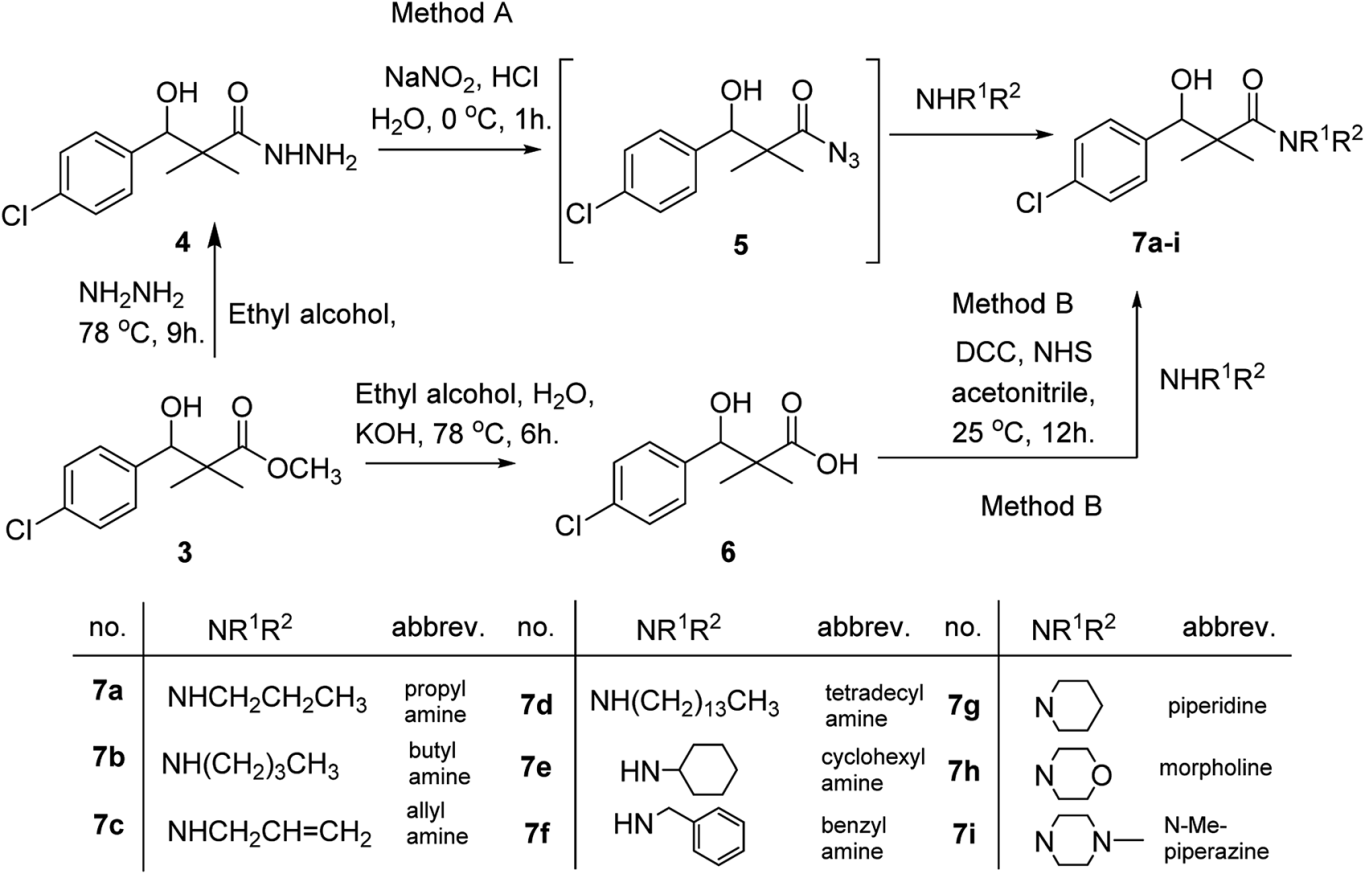
Synthesis of *N*-alkyl-3-(4-chlorophenyl)-3-hydroxy-2,2-dimethyl-propanamides 7a–i.

DCC and azide coupling methods are well-recognized in peptide synthesis centered on carboxylic acid-carbonyl group activation to be attacked by nucleophiles as amines or even weaker nucleophiles as amino acids to form a peptide bond.^10–13^ Structure modification of ester 3 could be achieved by attachment of alkane amine or amino acids to the carbonyl group of 3*via* DCC or azide coupling and the formation of peptide bond. Thus, the reaction of methyl 3-(4-chlorophenyl)-3-hydroxy-2,2-dimethylpropanoate (3) with hydrazine hydrate in ethanol under reflux condition for 9 h afforded the corresponding hydrazide 4. The model ester 3 was hydrolyzed using KOH solution in 50% alcohol water mixture at 78 °C for 6 h to afford the corresponding carboxylic acid 6, Scheme 2.

Hydrazide 4 was reacted with NaNO_2_ and HCl in water at 0 °C for 1 h to afford the corresponding azide 5 and were extracted with ethyl acetate. The *in situ* generated azide 5 solution was successively added to primary amines; propyl amine, butyl amine, allyl amine, tetradecyl amine, cyclohexyl amine and benzyl amine or secondary amines; piperidine, morpholine and *N*-methylpiperazine at 0 °C for 12 h to afford the corresponding *N*-alkyl-3-(4-chlorophenyl)-3-hydroxy-2,2-dimethylpropanamides 7a–i following the azide coupling method, Scheme 2.

The reaction of carboxylic acid derivatives 6 with primary amines and secondary amines in the presence of *N*,*N*′-dicyclohexylcarbodiimide and *N*-hydroxylsuccinimide (NHS) in acetonitrile at room temperature for 12 h afforded our products 7a–i as an equivocal method of preparation following HOSu-DCC coupling method, Scheme 2. Comparing both coupling methods leading to 7a–i we found out that azide coupling was more efficient respect to % of yield and simple reaction workup.

The structure assignment of the prepared *N*-alkyl-3-(4-chlorophenyl)-3-hydroxy-2,2-dimethylpropanamides 7a–i is based on ^1^H and ^13^C NMR spectral and physicochemical analysis. The ^1^H NMR spectrum of 3-(4-chlorophenyl)-3-hydroxy-2,2-dimethyl-*N*-propylpropanamide 7a shows signals at *δ* 5.72, 4.68, 4.07 and 4.05–4.02 ppm corresponding to NH, OCH, OH and CH_2_ groups respectively. The ^13^C NMR spectrum of 7a shows signals at 178.9, 80.9, 60.8 ppm (C=O), (OCH) and (C) groups respectively.

**Scheme 3:**
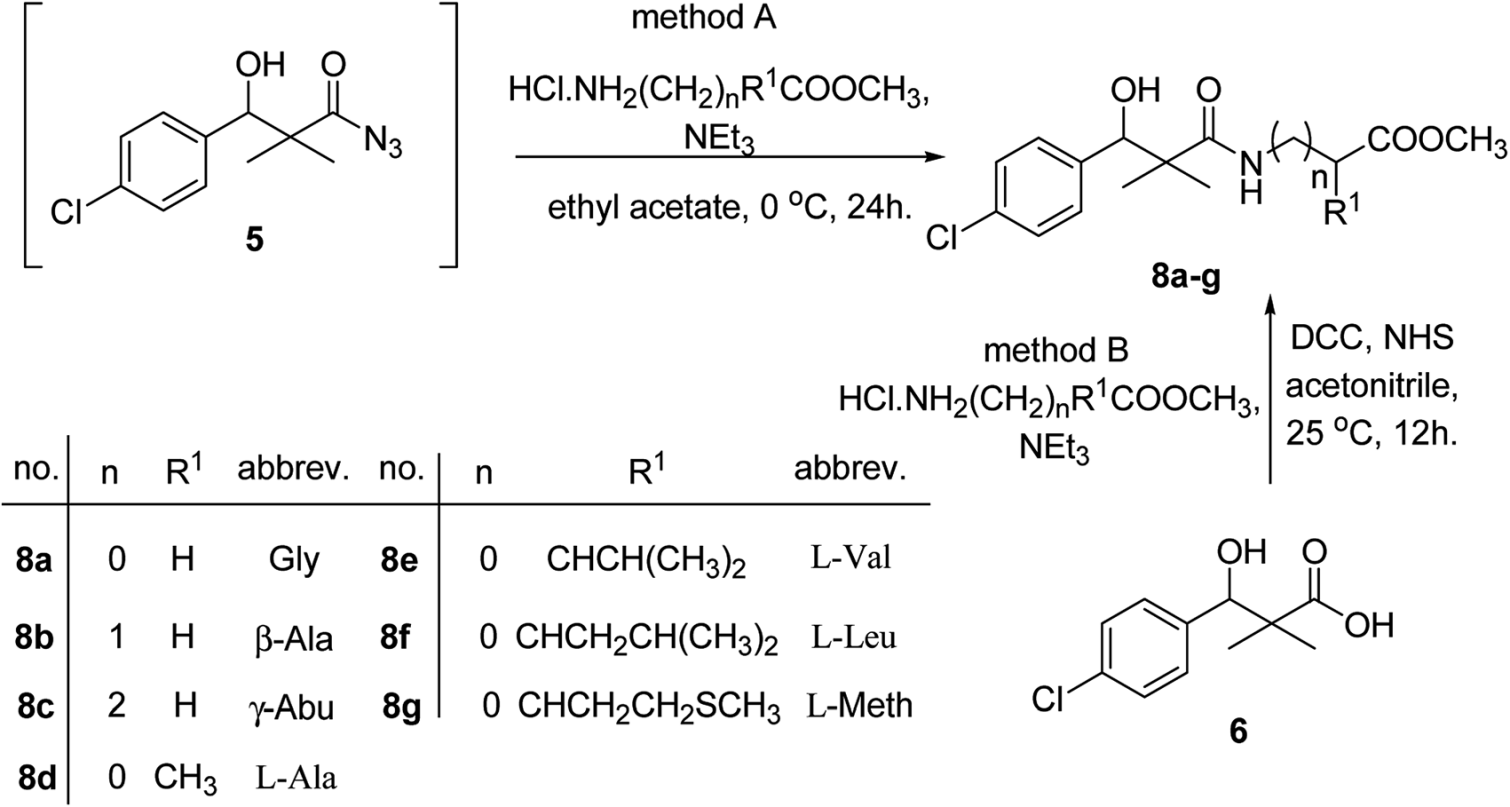
Synthesis of methyl-2-[(3-(4-chlorophenyl)-3-hydroxy-2,2-dimethylpropanoyl)amino]alkanoates 8a–g.

Similarly, methyl-2-[(3-(4-chlorophenyl)-3-hydroxy-2,2-dimethylpropanoyl)amino]alkanoates 8a–g were prepared by the reaction of the *in situ* generated azide 5 solution in ethyl acetate with amino acid ester hydrochlorides; glycine, β-alanine, γ-amino butyric acid, l-alanine, l-valine, l-leucine and l-methionine in the presence of triethylamine to give 8a–g in excellent yields, Scheme 3. An equivocal synthesis of 8a–g was achieved by the reaction of acid 6 with amino acid ester hydrochlorides in the presence of *N*,*N*′-dicyclohexylcarbodiimide and *N*-hydroxylsuccinimide (NHS) in acetonitrile at room temperature for 12 h afforded our product 8a–g, Scheme 3.

The structure assignment of the prepared methyl-2-[(3-(4-chlorophenyl)-3-hydroxy-2,2-dimethylpropanoyl)amino]alkanoates 8a–g is based on ^1^H and ^13^C NMR spectral and physicochemical analysis. The ^1^H NMR spectrum of methyl 2-[(3-(4-chlorophenyl)-3-hydroxy-2,2-dimethylpropanoyl)amino]acetate 8a shows signals at *δ* 6.67, 5.22, 4.63, 3.92 and 3.68 ppm corresponding to NH, OH, CH, NCH_2_ and OCH_3_ groups, respectively. The ^13^C NMR spectrum of 8a shows signals at 177.6, 170.1, 79.7, 51.5, 45.2, 41.4 (C=O), (C=O), (OCH), (OCH_3_), (C) and (NCH_2_) groups, respectively.

**Scheme 4:**
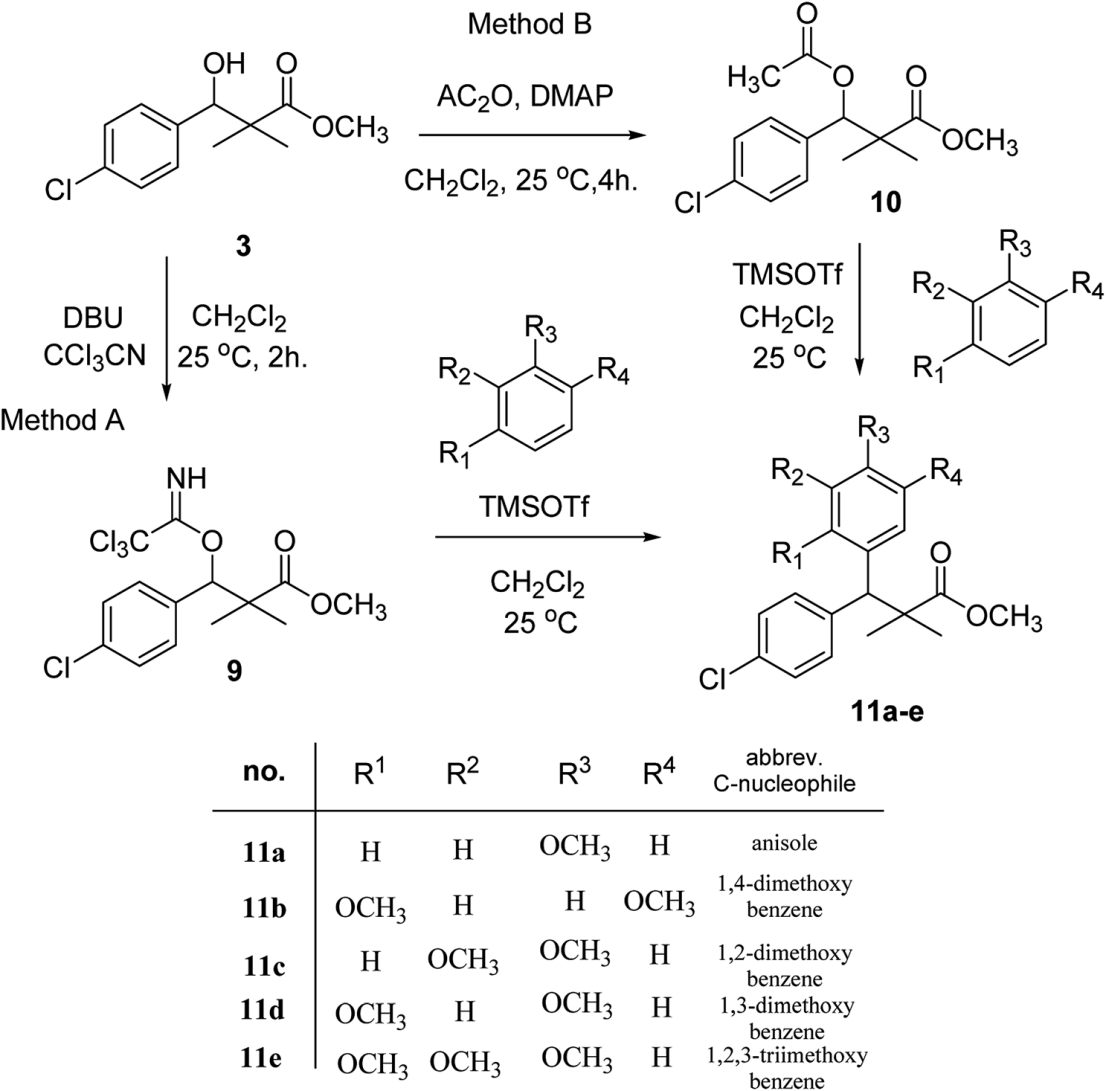
Synthesis of methyl-3-aryl-3-(4-chlorophenyl)-2,2-dimethylpropanoate 11a–e.

Trichloroacetimidate and acetate C–C coupling proved to be excellent methods for structure modifications of alcohols.^14–18^ These methods are related to transform hydroxyl group substrates into the appropriate trichloroacetimidate or acetate as excellent leaving groups by the reaction with trichloroacetonitrile or acetic anhydride, respectively. The successive addition of C-nucleophiles mainly activated arenes, allyltrimethylsilane and trimethylsiloxyalkenes to the active trichloroacetimidate or acetate intermediates in the presence of Lewis acid gave the desired products through the formation of new C–C bond.^14–18^ We find it interesting to apply the trichloroacetimidate and acetate coupling methods in the structure modification of methyl-3-(4-chlorophenyl)-3-hydroxy-2,2-dimethylpropanoate (3) *via* C–C coupling with methoxybenzene derivatives. Thus, the reaction of the ester 3 with trichloroacetonitrile in presence of DBU in dichloromethane at 25 °C for 2 h afforded the trichloroacetimidate 9. Similarly, the reaction of ester 3 with acetic anhydride in the presence of DMAP (*N*,*N*-dimethylaminopyridine) in dichloromethane at 25 °C for 4 h afforded methyl 3-acetoxy-3-(4-chlorophenyl)-2,2-dimethylpropanoate (10). The reaction of trichloroacetimidates 9 or acetate 10 with arene C-nucleophiles; anisole, 1,4-dimethoxybenzene, 1,2-dimethoxybenzene and 1,2,3-trimethoxybenzene in the presence of catalytic amount of trimethylsilyltrifluoro-methanesulfonate (TMSOTf) (0.1 eq.%) at room temperature gave readily the methyl 3-aryl-3-(4-chlorophenyl)-2,2-dimethylpropanoate 11a–e in excellent yields, Scheme 4.

Comparing the efficiency of both C–C coupling methods according to reaction time and percent of yield, results showed that, although, all compounds were prepared in good yields, there was a slight improvement in the percent of yield and in the reaction time (monitored by TLC) using trichloroacetimidate method, Table 1.

**Table 1:** Comparing the efficiency of trichloroacetimidate and acetate coupling methods

No.	Structure	Yields (isolated)	No.	Structure	Yields (isolated)
11a	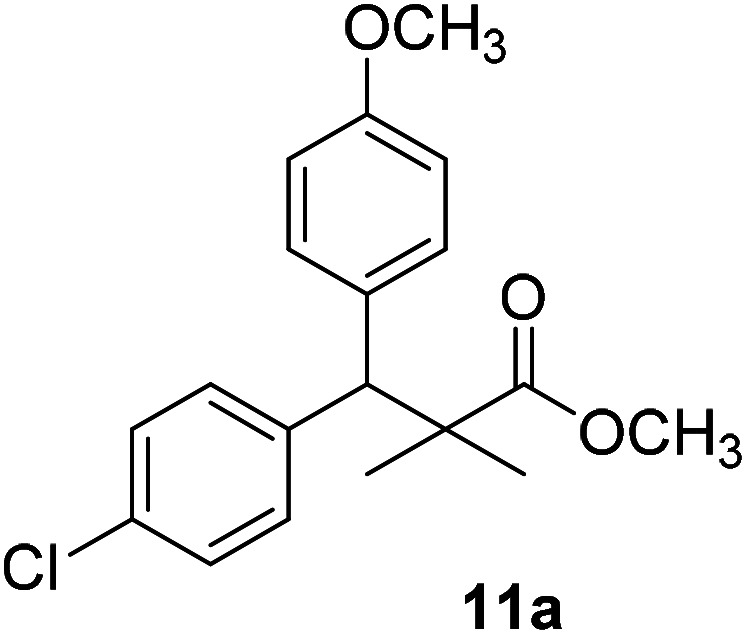	84%^a^, 77%^b^, 2 h	11b	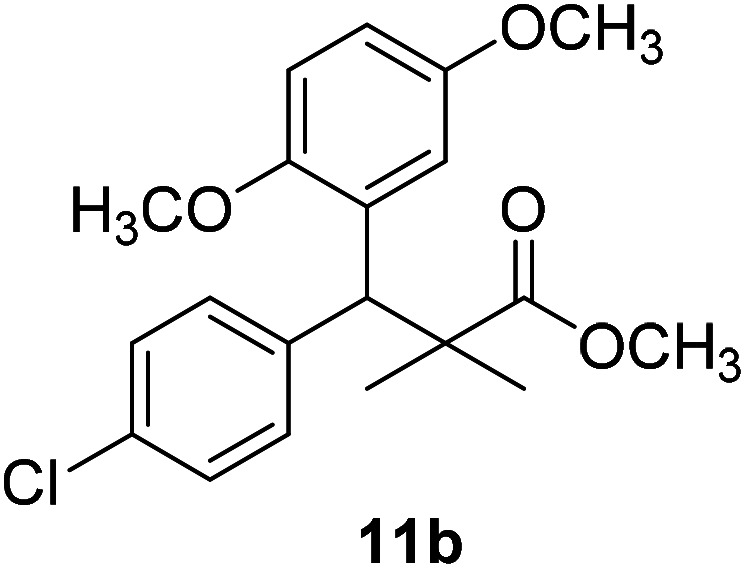	81%^a^, 76%^b^, 2 h
11c	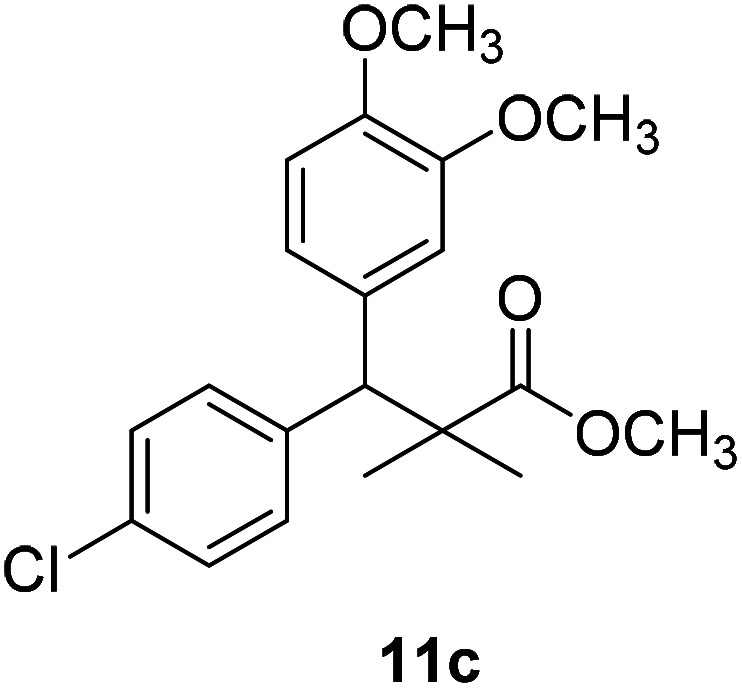	78%^a^, 65%^b^, 2 h	11d	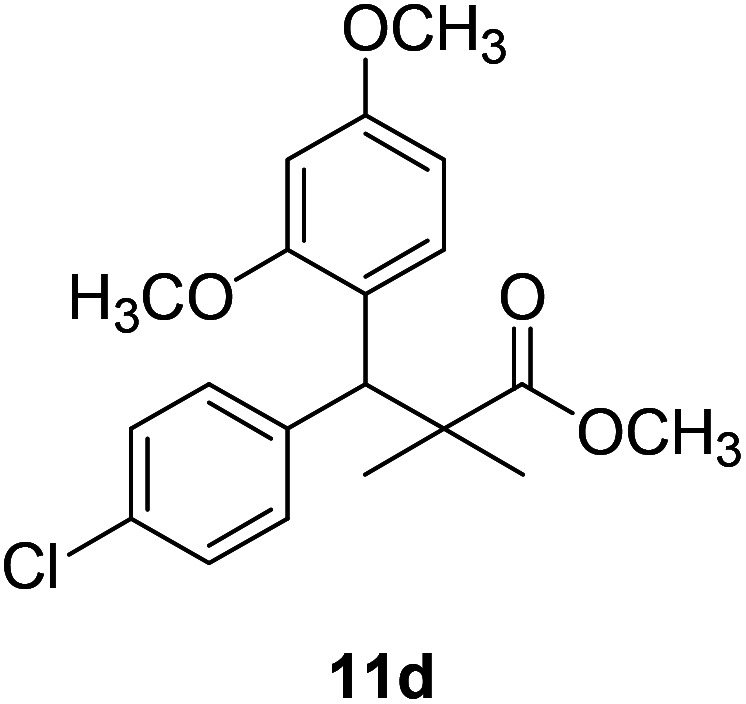	80%^a^, 69%^b^, 2 h
11e	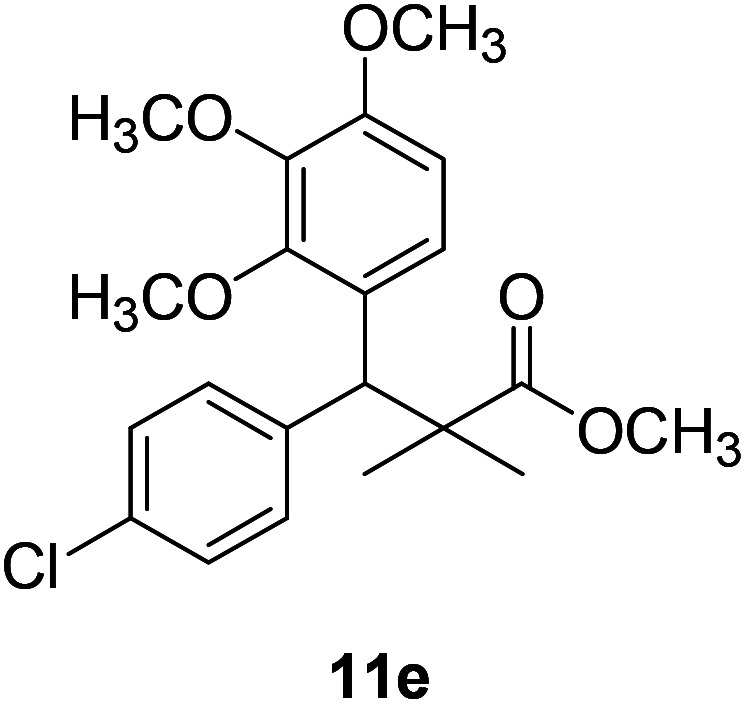	88%^a^, 74%^b^, 2 h			

a Yield respect to trichloroacetimidate coupling method.

b Yield respect to acetate coupling method.

The structure assignment of the prepared methyl 3-aryl-3-(4-chlorophenyl)-2,2-dimethylpropanoate 11a–e is based on ^1^H and ^13^C NMR spectral and physicochemical analysis. The ^1^H NMR spectrum of methyl 3-(4-chlorophenyl)-3-(4-methoxyphenyl)-2,2-dimethylpropanoate (11a) shows signals at *δ* 4.23, 3.68, 3.44 ppm corresponding to CH, OCH_3_ and OCH_3_ groups, respectively. The ^13^C NMR spectrum of 11a shows signals at *δ* 176.2, 56.1, 53.2, 48.5, 47.2 ppm corresponding to (C=O), (OCH_3_), (OCH_3_), (CH) and (C) groups, respectively.

### Antiproliferative activities

The cytotoxic impact of compounds on cancer cell's viability was assessed by MTT assay. The 48 post-treatments showed that out of 24 compounds, 12 compounds showed inhibitory actions on HCT-116 cells, whereas 10 compounds did not show any inhibitory actions. We have calculated the inhibitory action (IC_50_) of these compounds on both HCT-116 and HEK-293 cells and we have found that IC_50_ were in between is 0.12 mg mL^−1^ to 0.81 mg mL^−1^. The compounds (7a, 7g) showed highest inhibitory activity (0.12 mg mL^−1^), whereas compound 7d showed lowest inhibitory activity (0.81 mg mL^−1^). We have also examined inhibitory action on normal and non-cancerous cells (HEK-293 cells) to confirm whether these compounds produce any effects on them. We have tested these compounds on normal healthy cells (HEK-293) and MTT results revealed that all these compounds did not produce any inhibitory action on the normal cells which suggest that synthetized compounds are specially targeted to cancerous cells. There are several reports of treatments of biomaterials which showed inhibitory action on different cancerous cells.^19–27^ The IC_50_ values for all compounds were given in the Table 2.

**Table 2:** Antiproliferative activity of synthetic compounds^a^

Compound number	Structure of compounds	IC_50_ value HCT-116	IC_50_ value HEK-293
4	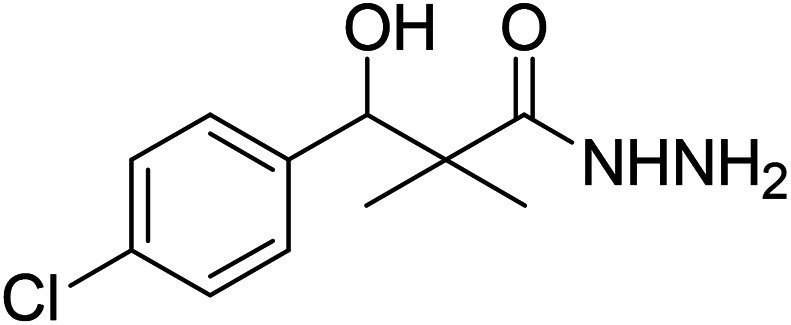	0.41 mg mL^−1^	NA
6	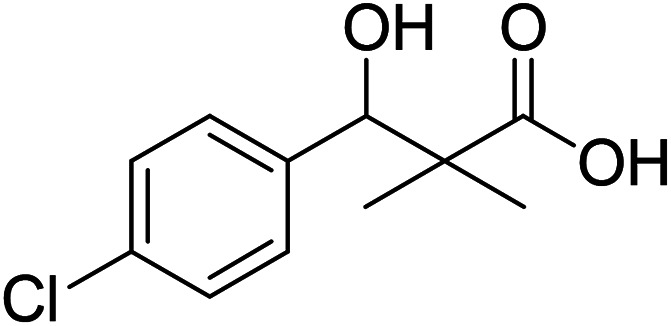	0.29 mg mL^−1^	NA
7a	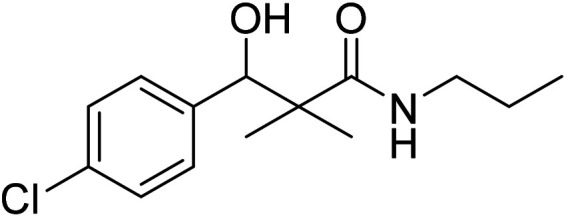	0.12 mg mL^−1^	NA
7b	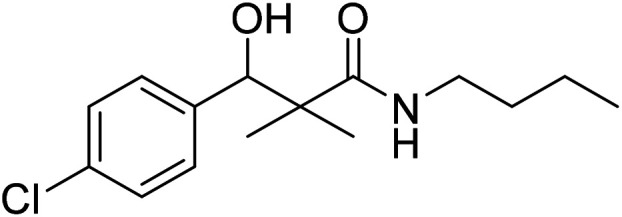	NA	NA
7c	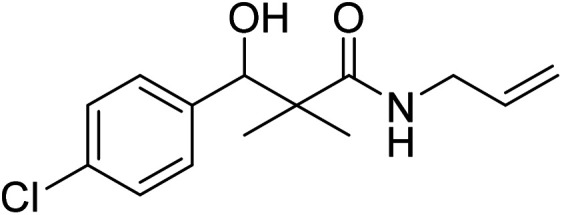	0.22 mg mL^−1^	NA
7d	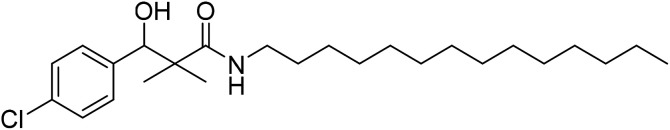	0.81 mg mL^−1^	NA
7e	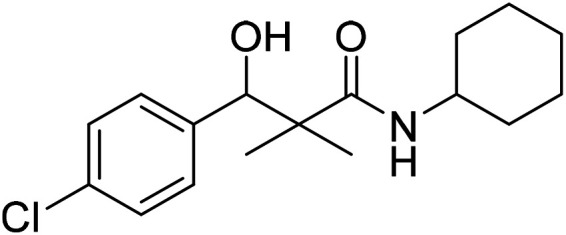	NA	NA
7f	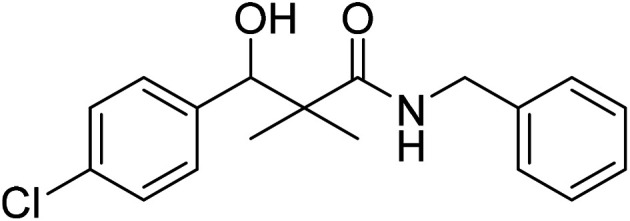	0.61 mg mL^−1^	NA
7g	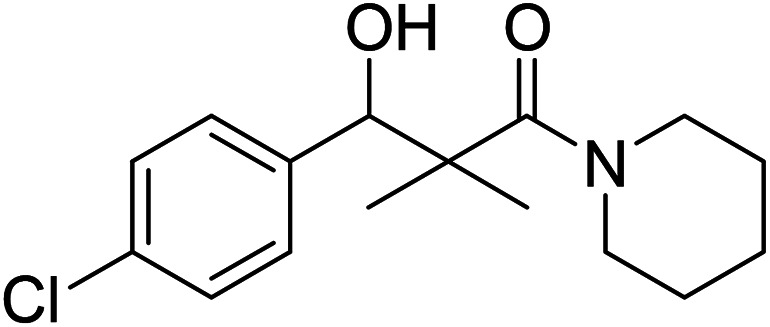	0.12 mg mL^−1^	NA
7h	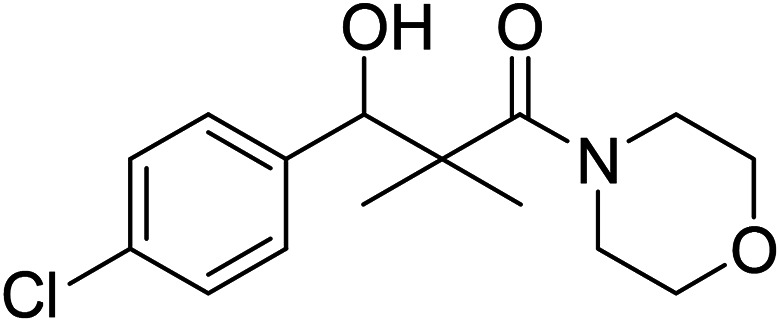	0.68 mg mL^−1^	NA
7i	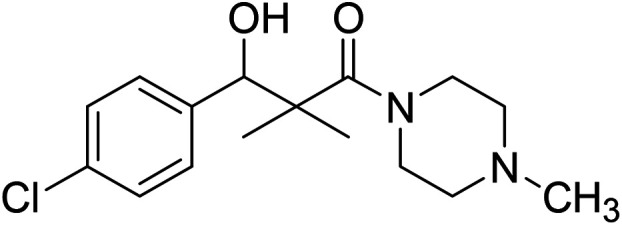	0.13 mg mL^−1^	NA
8a	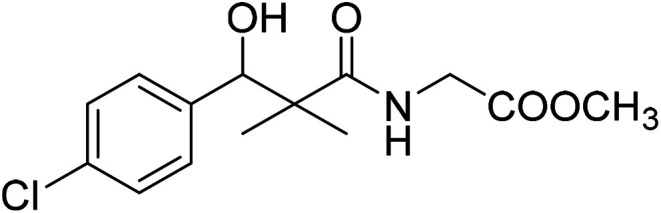	0.51 mg mL^−1^	NA
8b	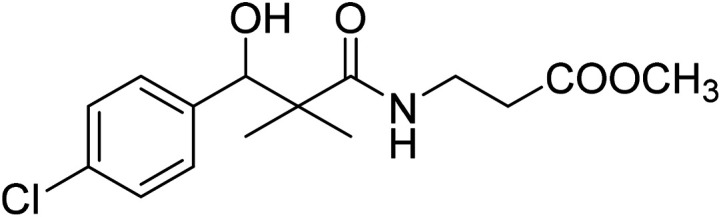	0.58 mg mL^−1^	NA
8e	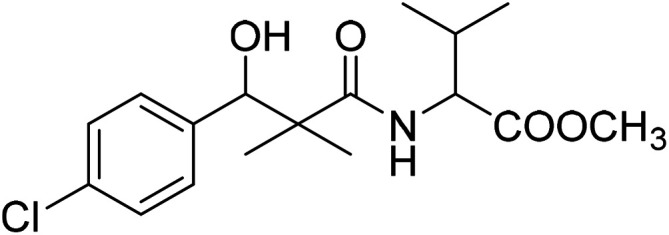	NA	NA
8f	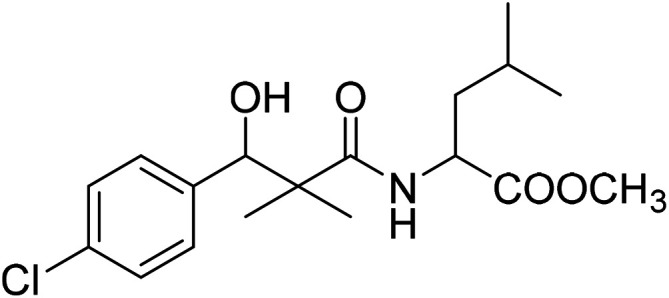	0.71 mg mL^−1^	NA
8g	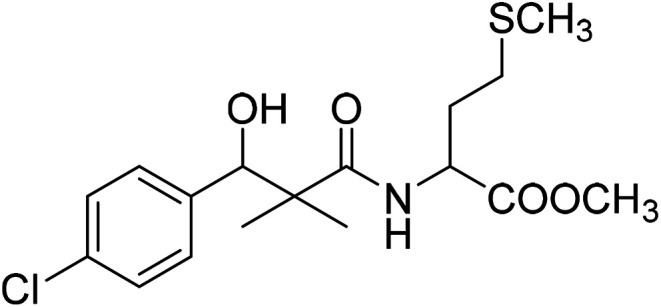	NA	NA
10	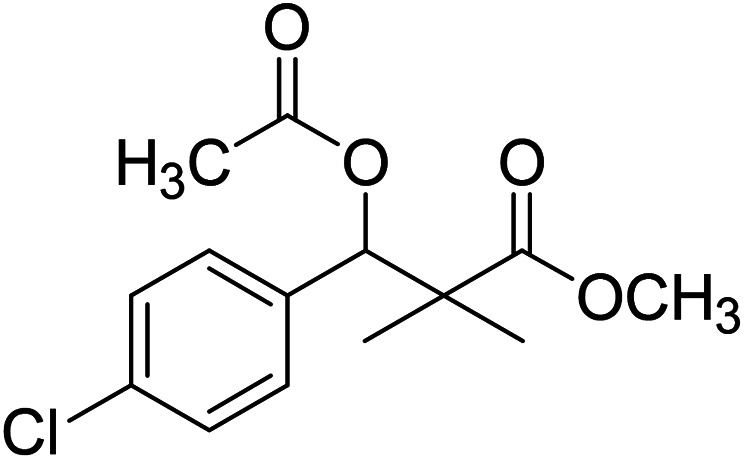	NA	NA
11a	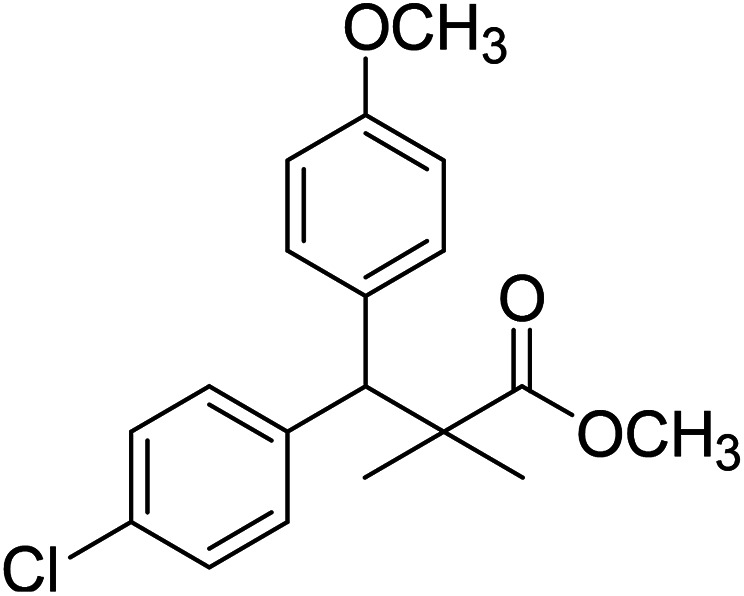	NA	NA
11b	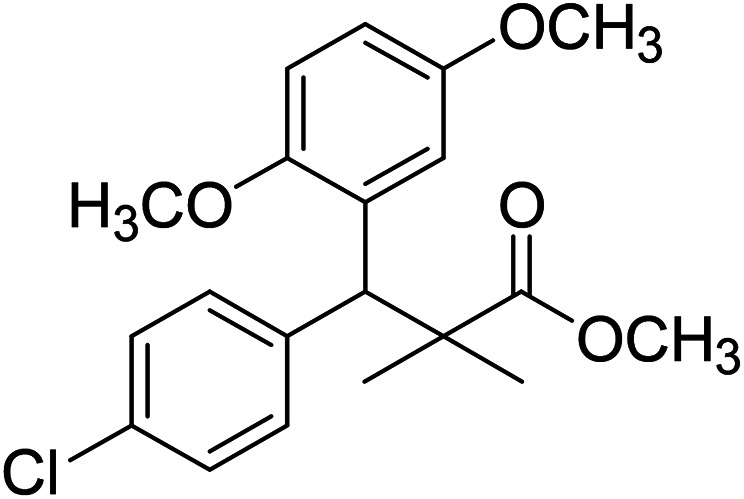	NA	NA
11c	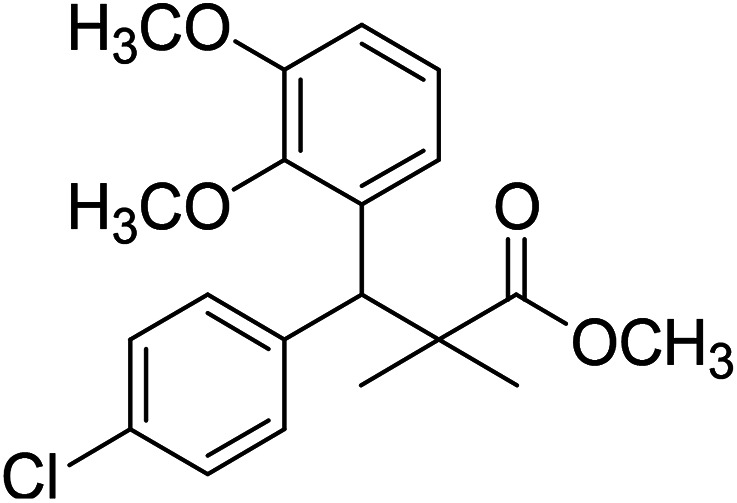	NA	NA
11d	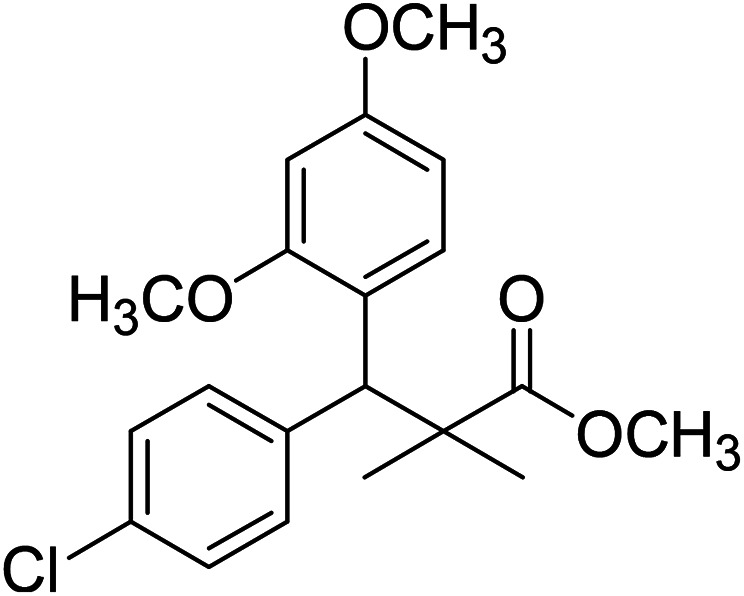	NA	NA
11e	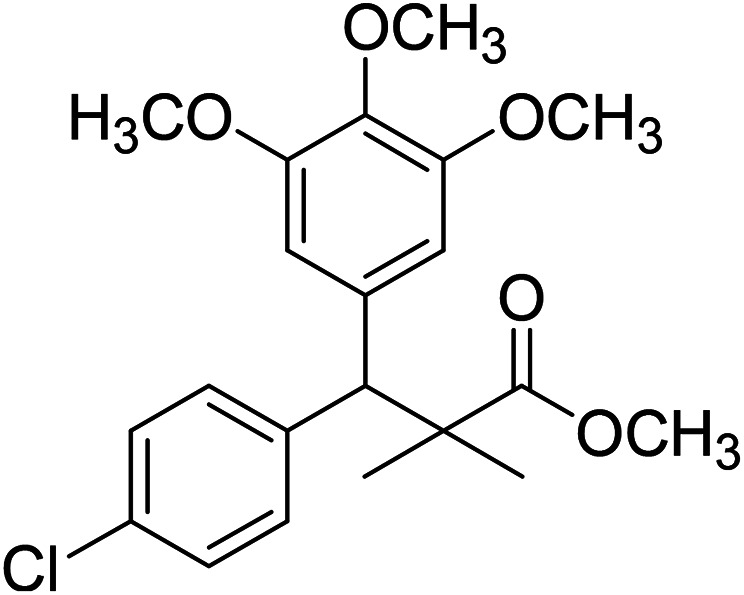	NA	NA

a NA = not active, IC_50_ value [mg mL^−1^] = inhibitory concentration (IC) is expressed in mg mL^−1^.

The preliminary structure–activity relationship of the compounds showed that all active compounds contain the free OH on the α carbon (4, 6, 7a, 7c, 7d, 7f, 7g, 7h, 7i, 8a, 8b, 8f), while most of the inactive compounds lack the OH (10, 11a, 11b, 11c, 11d, 11e). All compounds having esters alpha to the isopropyl group are inactive (10, 11a, 11b, 11c, 11d, 11e) while those with an acid or substituted amides are active (4, 6, 7a, 7c, 7d, 7f, 7g, 7h, 7i, 8a, 8b, 8f). This could be partly due to that all the compounds with ester functionality lacks the free OH on the α carbon. Substitution on the amide is crucial; with compounds having bulky branched chain are inactive (8e, 8g). Electron rich amides are more favorable as they can participate better in hydrogen bonding as acceptor (7g and 7i compared to 7h). Therefore, a hydrogen donor group linked to the halo substituted ring is essential. A hydrogen acceptor group such as an acid or alkyl substituted amides are important for activity. Substitution on the amide is detrimental. The amide nitrogen could be a part of a heterocyclic ring however oxygen containing heterocycles are less active due to the higher electronegativity and electron withdrawing properties of the oxygen (7g and 7i compared to 7h).

### Nuclear disintegration

**Fig. 1:**
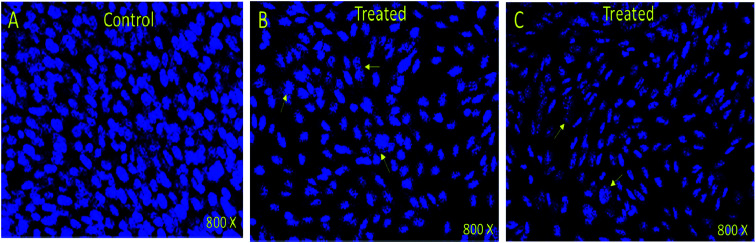
DAPI stained HCT-116 cells. (A) Control (nontreated) (B) treated (7i) compound (0.13 mg mL^−1^) and (C) treated with (7g) compound (0.12 mg mL^−1^). Nuclear disintegration, and chromatic condensation are observed (arrows) in the (B) and (C). 800× magnifications.

In order to understand whether cancer cell death is due to necrosis or apoptosis, we have stained cancer cells with DAPI (4′,6-diamidine-2′-phenylindole dihydrochloride), which is considered as the marker for the apoptosis.^29–32^ We have observed clear DNA disintegration and fragmentation in the cells treated with compounds (7i and 7g) with compared to untreated or control cells (Fig. 1B and C). In addition, we have observed condensed chromatins and nuclear fragmentations in cancerous cells which is not observed in the untreated (control) cells (Fig. 1A). The nuclear disintegration of cancerous cells is due to programmed cell death and similar results were also observed in other studies.^21,22,28^

**Fig. 2:**
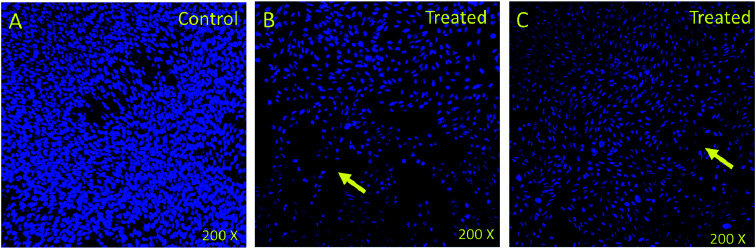
DAPI stained HCT-116 cells. (A) Control (nontreated) (B) treated (7i) compound (0.13 mg mL^−1^) and (C) treated with (7g) compound (0.12 mg mL^−1^). DAPI stained cells visualized through confocal microscope and (B) and (C) show significant loss (arrows) of staining due to treatment. 200× magnifications.

We have also observed decrease in DAPI staining in the compound-treated cells compared to untreated cells, which suggest that compounds inhibited the growth of the cancer cells as observed in other studies (Fig. 2).^33^

### Molecular modeling

The mechanism of action of the compounds antiproliferative and apoptotic activity were investigated using molecular docking experiments. Heat shock protein 90 (Hsp90) is a molecular chaperone, which plays a key role in signal transduction pathways, whose inhibition has been widely described as one of the potential cancer's treatments.^34^ Tumor necrosis factor receptor-associated protein 1 (TRAP1), a member of the HSP90 family, is involved in several physiological functions, including cell proliferation, differentiation, and survival. The anti-apoptotic roles of TRAP1 were explored as an important target for anticancer drug development and for tumor control in clinical settings.^34^ Therefore, the mitochondrial pool of Hsp90 and its mitochondrial paralogue, TRAP1, have been studied as target proteins for anticancer drug development.^35^

**Fig. 3:**
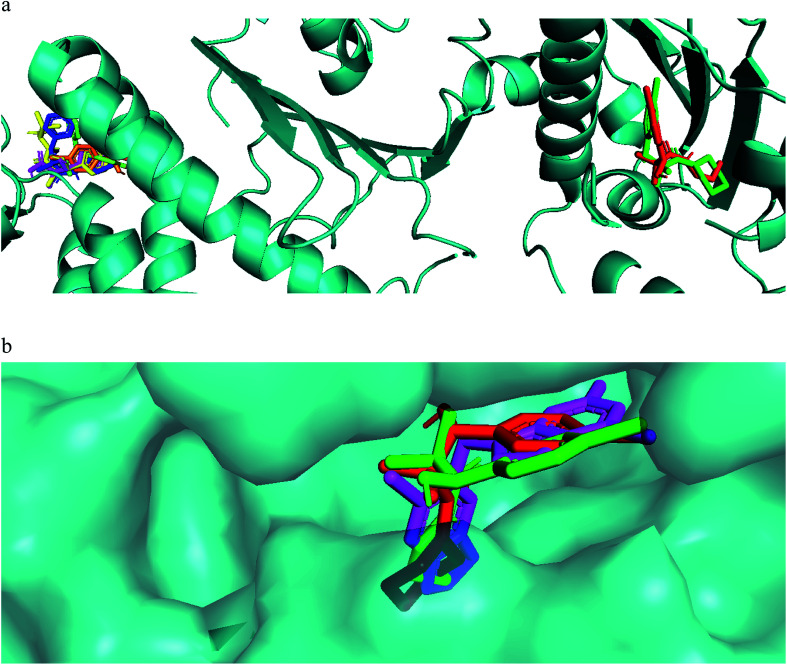
(a) Compound 7a (red) and 7g (green) docked in the ATP binding site of TRAP1, while less active compounds 6 (magenta), 7e (blue), 8a (brown), 8e (yellow) docked in a pocket far away from the binding site. (b) Molecular surface representation of compound 7a (red), compound 7b (green), and co crystallized ligand (magenta) in TRAP1 active site (front view).

Our compounds showed affinity to the heat shock proteins TRAP1 *in silico* and this could be a potential mode of action that explain the compounds antiproliferative activities. Hsp90 inhibitors anticancer activity has been found modest compared to their TRAP1 inhibitors counterpart.^36^ Therefore, inhibitors with strong TRAP1-binding but weak Hsp90-binding, activities would have better *in vivo* activities and clinical applications. Interestingly, the most active compounds 7a, 7g docked well in the active site of TRAP1 while compounds with moderate activity or inactive were docked far away from the active site. The most active compounds showed also lower binding affinity to HSP90. They lacked the hydrogen bonding interactions with Asp93 and Thr184 that was involved in a network of hydrogen bonding in case of 8a and the crystallized ligand. This when added to their high affinity to TRAP1 could give an indication that this is a potential mode of action for further research. The main difference in the TRAP1 active site structure compared to HSP90 were reported to be in the active site lid (Leu172–Phe201) and its flanking conserved residues, Asn171 and Gly202, that have different configurations in TRAP1 compared with those in Hsp90. Only compounds 7a and 7g, the most active ones, were found in the ATP binding site of TRAP1 (Fig. 3a). Comparative docking revealed that those compounds showed docking poses that is almost identical to the co-crystallized inhibitor (Fig. 3b). The surface representation showed that they have their side chain with amide and alcohol functional groups buried deeply in the hydrophobic ATP pocket and their aromatic ring directed toward the solvent exposed surface (Fig. 3b).

**Fig. 4:**
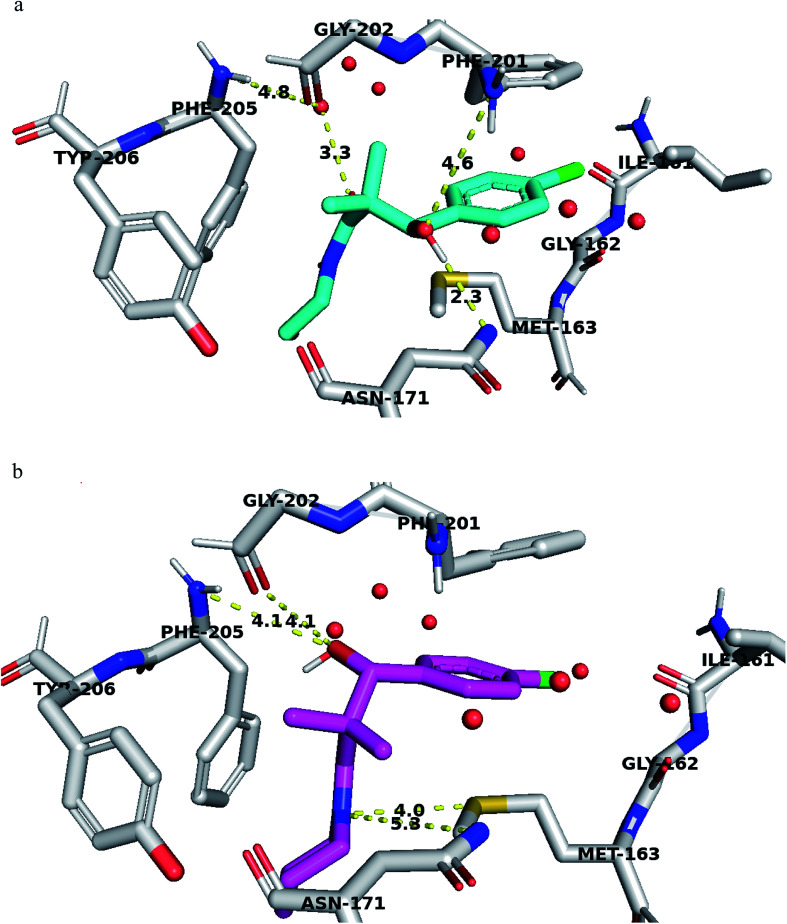
(a) Compound 7a docked in TRAP1 ATP binding site and performing interaction with key residues. (b) Compound 7g docked in TRAP1 ATP binding site and performing interaction with key residues. Distances are represented as yellow dotted lines in angstrom.

Structural analyses of the docking poses revealed that both 4-chlorophenyl ring of 7a and 7b are relatively close to the flanking regions of the disordered TRAP1 active site lid and established additional π–π stacking interactions with the conserved Phe201 residue in TRAP1 (Fig. 4).

Furthermore, 7a exhibited superior binding affinity to TRAP1 through hydrogen bonding between the side chain OH and the Asn171 side chain amide and another potential hydrogen bond with Phe201 (Fig. 4a). An additional water-mediated hydrogen bond was also noted with Phe205 side chain.

Compound 7g showed potential hydrogen bonds of its side chain amide nitrogen with both Asn171 side chain amide and Met163 side chain. The compound side chain OH exhibited potential hydrogen bonds with Gly202 and Phe205 (Fig. 4b).

Thus, the TRAP1 selective binding of 7a and 7g was explained based on their superior and characteristic interactions with Asn171 and Phe201, located on the flanked residues of the disordered TRAP1 structure.

The binding affinity of these compounds was found consistent with their superior TRAP1 activity. Binding affinity calculation showed that compounds 7a and 7g had the best affinity to TRAP1 among all other compounds with values of −7.6 and −7.5 kcal mol^−1^ for 7a and 7g respectively.

Insufficient mitochondrial penetration of Hsp90 inhibitors is potentially due to a strong binding to abundant (∼2% of total cellular proteins) cytoplasmic Hsp90 proteins.^31^

**Fig. 5:**
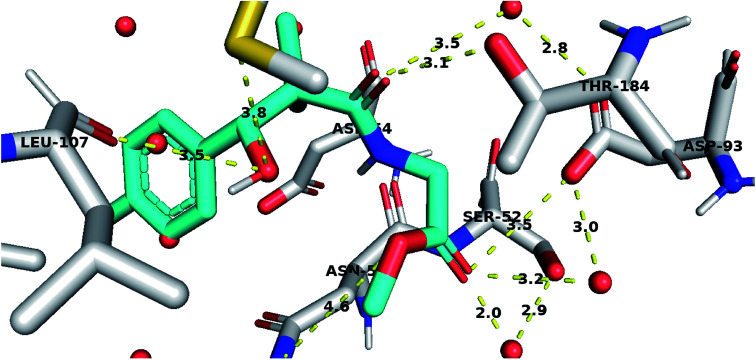
Compound 8a docked in HSP90 ATP binding site and performing interaction with key residues. Distances are represented as yellow dotted lines in angstrom.

To further test the activity of the less active compounds they were docked in the cytoplasmic HSP90. Inhibitors of HSP90 are known to bind to an ATP binding pocket in the *N*-terminal domain of the protein. Compound 8a displayed a binding mode similar to other known Hsp90 inhibitors. The side chain occupied the adenosine pocket and was involved in a network of hydrogen bonding. The terminal ester carbonyl oxygen made key interactions with Asp93 both through direct hydrogen bonding and through a conserved water molecule while the amide oxygen formed a water-mediated hydrogen bond with Asp93. The side chain amide oxygen also established hydrogen bond with Thr184. The 4-chlorophenyl group was stacked between the side chain of Leu107 and Phe138 and was held tightly in the hydrophobic pocket adjacent to the adenosine binding site through a bidentate hydrogen bond interaction with Leu107 and Met98 (Fig. 5).

TRAP1 and HSP90 have been found to be elevated in cancer cells where these proteins suppress cell death. This finding could partly explain the selectivity shown by those compounds and their inactivity on normal cells. Therefore, the herein described compounds being working on more selective targets have higher chances to be developed to clinically effective agents after the proper pharmacodynamic and pharmacokinetic optimization.

## Experimental

### General procedures

Solvent were purified and dried in the usual way. The boiling range of the petroleum ether used was 40–60 °C. Thin layer chromatography (TLC): silica gel 60 F_254_ plastic plates (E. Merck, layer thickness 0.2 mm) detected by UV absorption. Melting points were determined on a Buchi 510 melting-point apparatus and the values are uncorrected. ^1^H and ^13^C NMR spectra were recorded at 400 MHz and 100 MHz, respectively (Bruker AC 400) in CDCl_3_ and DMSO solution with tetramethylsilane as an internal standard. The NMR analyses were performed at Faculty of Science, Sohag University. The mass spectra were measured with a KRATOS analytical compact; on MALDI-MS the spectrometer was using 2,5-dihydroxy benzoic acid (DHB) as matrix. The starting compound 3 were obtained as described in literature.^37,38^

### Preparation of 3-(4-chlorophenyl)-3-hydroxy-2,2-dimethylpropanoic acid hydrazide (4)

To a solution of methyl 3-(4-chlorophenyl)-3-hydroxy-2,2-dimethylpropanoate (3) (0.24 g, 1.0 mmol) in ethyl alcohol (10 mL), hydrazine hydrate (80%) (4.0 mmol) was added and the reaction mixture was heated under reflux for 9 h. The reaction mixture was cooled and evaporated under reduced pressure till dried. The precipitated residue was crystallized from ethyl alcohol. White crystals (89%), mp: 96–98 °C. ^1^H NMR spectrum, (400 MHz, CDCl_3_), *δ*, ppm (*J*, Hz): 8.70 (1H, bs, NH), 7.34 (2H, t, *J* = 8.0 Hz, ArH), 7.25 (2H, t, *J* = 8.0 Hz, ArH), 5.56 (1H, d, *J* = 4.0 Hz, CH), 4.77 (1H, d, *J* = 4.0 Hz, OH), 4.17 (2H, bs, NH_2_), 1.03 (3H, s, CH_3_), 0.90 (3H, s, CH_3_). (MALDI, positive mode, matrix DHB): *m*/*z* = 265 (M + Na)^+^.

### Preparation of 3-(4-chlorophenyl)-3-hydroxy-2,2-dimethylpropanoic acid (6)

To a solution of methyl 3-(4-chlorophenyl)-3-hydroxy-2,2-dimethylpropanoate (3) (0.24 g, 1.0 mmol) in 70% ethyl alcohol (10 mL), KOH (0.084, 1.5 mmol) and 2 mL H_2_O were added and the reaction mixture was heated under reflux for 6 h. The reaction mixture was cooled and acidification by dilHCl. The precipitated residue was crystallized from ethyl alcohol. White crystals (74%), mp: 171–173 °C. ^1^H NMR spectrum, (400 MHz, CDCl_3_), *δ*, ppm (*J*, Hz): 11.98 (1H, bs, OH), 7.37–7.31 (4H, m, ArH), 4.83 (1H, s, CH), 3.29 (1H, bs, OH), 1.03 (3H, s, CH_3_), 0.89 (3H, s, CH_3_). ^13^C NMR spectrum, (100.0 MHz, CDCl_3_), *δ*, ppm: 182.9 (C=O), 146.3, 136.8, 134.5, 132.5, 130.1, 127.4 (C-Ar), 81.2 (CH), 61.3 (C), 26.4 (CH_3_), 25.1 (CH_3_). (MALDI, positive mode, matrix DHB): *m*/*z* = 251 (M + Na)^+^.

### Preparation of *N*-alkyl-3-(4-chlorophenyl)-3-hydroxy-2,2-dimethylpropanamides 7a–i

Method A: to a cold solution (−5 °C) of 3-(4-chlorophenyl)-3-hydroxy-2,2-dimethylpropanoic acid hydrazide (4) (0.24 g, 1.0 mmol) in 1 N HCl solution (5 mL) and acetic acid 96% (2 mL), 5 mL water solution of NaNO_2_ (0.11 g, 1.5 mmol) was added. The reaction mixture was stirred at 0 °C for 30 min. The reaction mixture was extracted three times with 30 mL ethyl acetate. The combined yellow syrup extract was washed several times with 3% solution of NaHCO_3_ and water till it became neutral and was finally dried over Na_2_SO_4_. The *in situ* generated azide 5 was used without further isolation and purification. To the previously prepared cold ethyl acetate solution of azide 5, a solution of appropriate amines (1.0 mmol) in ethyl acetate (10 mL) was added dropwise with stirring. The reaction mixture was kept at −5 °C for 12 h, then at room temperature for another 12 h. The reaction mixture was washed with 0.5 N HCl, water and 3% solution of NaHCO_3_ and dried over Na_2_SO_4_. The solution was evaporated to dryness and the residue was recrystallized from ethyl acetate/petroleum ether to give the desired product 7a–i.

Method B: to a cold solution (−5 °C) of amines (1.0 mmol) and triethylamine (0.10 mL, 1.0 mmol) in acetonitrile (6 mL) was added 3-(4-chlorophenyl)-3-hydroxy-2,2-dimethylpropanoic acid (6) (0.23 g, 1.0 mmol), dicyclohexylcarbodiimide (DCC) (0.21 g, 1.0 mmol) and *N*-hydroxylsuccinimide (NHS) (0.10 g, 1.0 mmol), successively. The reaction mixture was stirred at 0 °C for one hour, at 5 °C for one hour and at room temperature for 12 h. The reaction mixture was set aside overnight. The precipitated dicyclohexyl urea was filtered off and the filtrate was evaporated under reduced pressure. The residue was purified by silica gel column chromatography eluted with EtOAc/petroleum ether 1 : 4 to give pure 7a–i.

### 3-(4-Chlorophenyl)-3-hydroxy-2,2-dimethyl-*N*-propylpropanamide (7a)

White crystals (81%), mp: 186–188 °C. ^1^H NMR spectrum, (400 MHz, CDCl_3_), *δ*, ppm (*J*, Hz): 7.27–7.20 (4H, m, ArH), 5.72 (1H, bs, NH), 4.68 (1H, s, CH), 4.07 (1H, bs, OH), 4.05–4.02 (2H, m, CH_2_), 1.39–1.20 (2H, m, CH_2_), 1.13 (3H, s, CH_3_), 1.09 (3H, t, *J* = 4.0 Hz, CH_3_), 0.90 (3H, s, CH_3_). ^13^C NMR spectrum, (100.0 MHz, CDCl_3_), *δ*, ppm: 178.9 (C=O), 136.8, 133.4, 132.9, 130.5, 128.3, 127.1 (C-Ar), 80.9 (CH), 60.8 (C), 34.5 (CH_2_), 23.6 (CH_2_), 23.4 (CH_3_), 22.2 (CH_3_), 12.1 (CH_3_). (MALDI, positive mode, matrix DHB): *m*/*z* = 292 (M + Na)^+^.

Method B, from 6 and propylamine (74%).

### *N*-Butyl-3-(4-chlorophenyl)-3-hydroxy-2,2-dimethylpropanamide (7b)

White crystals (78%), mp: 75–76 °C. ^1^H NMR spectrum, (400 MHz, CDCl_3_), *δ*, ppm (*J*, Hz): 8.04–8.02 (1H, m, ArH), 7.93–7.90 (1H, m, ArH), 7.76–7.55 (3H, m, ArH, NH), 5.97 (1H, bs, OH), 4.62–4.57 (1H, m, CH), 3.53–3.47 (2H, m, CH_2_), 2.68–2.64 (2H, m, CH_2_), 1.58–1.41 (2H, m, CH_2_), 0.86 (3H, s, CH_3_), 0.84–0.81 (6H, m, 2CH_3_). ^13^C NMR spectrum, (100.0 MHz, CDCl_3_), *δ*, ppm: 175.2 (C=O), 139.9, 131.5, 128.4, 128.3, 127.8, 127.7 (C-Ar), 80.1 (CH), 54.2 (C), 39.8 (CH_2_), 33.5 (CH_2_), 21.3 (CH_3_), 20.1 (CH_3_), 19.2 (CH_2_), 13.8 (CH_3_). (MALDI, positive mode, matrix DHB): *m*/*z* = 306 (M + Na)^+^.

Method B, from 6 and butylamine (58%).

### *N*-Allyl-3-(4-chlorophenyl)-3-hydroxy-2,2-dimethylpropanamide (7c)

White crystals (79%), mp: 127–129 °C. ^1^H NMR spectrum, (400 MHz, CDCl_3_), *δ*, ppm (*J*, Hz): 7.23–7.14 (5H, m, ArH, NH), 5.92 (1H, bs, OH), 5.76–5.69 (1H, m, CH), 5.08 (1H, s, CH), 4.64 (2H, m, CH_2_), 3.86–3.60 (2H, m, CH_2_), 1.12 (3H, s, CH_3_), 1.01 (3H, s, CH_3_). ^13^C NMR spectrum, (100.0 MHz, CDCl_3_), *δ*, ppm: 179.2 (C=O), 148.4, 137.8, 135.4, 134.5 (CH), 132.9, 130.6, 127.4 (C-Ar), 117.3 (CH_2_), 81.2 (CH), 61.4 (C), 44.2 (CH_2_), 20.2 (CH_3_), 19.4 (CH_3_). (MALDI, positive mode, matrix DHB): *m*/*z* = 290 (M + Na)^+^.

Method B, from 6 and allylamine (65%).

### 3-(4-Chloro-phenyl)-3-hydroxy-2,2-dimethyl-*N*-tetradecyl-propanamide (7d)

White crystals (64%), mp: 89–91 °C. ^1^H NMR spectrum, (400 MHz, CDCl_3_), *δ*, ppm (*J*, Hz): 7.39–7.23 (4H, m, ArH), 6.07 (1H, bs, OH), 4.64 (1H, s, CH), 3.39–3.32 (2H, m, NCH_2_), 1.62–1.57 (4H, m, 2CH_2_), 1.29–1.19 (16H, m, 8CH_2_), 1.24–1.22 (2H, m, CH_2_), 1.04 (3H, s, CH_3_), 0.99–0.97 (3H, m, CH_3_), 0.91 (3H, s, CH_3_). ^13^C NMR spectrum, (100.0 MHz, CDCl_3_), *δ*, ppm: 178.6 (C=O), 139.3, 134.5, 129.4, 128.7, 127.3, 127.0 (C-Ar), 79.6 (CH), 45.9 (NCH_2_), 44.6 (C), 34.2 (CH_2_), 26.4 (CH_2_), 26.0 (CH_2_), 24.3 (CH_2_), 20.5 (CH_3_), 19.5 (CH_3_), 17.7 (CH_3_). Method B, from 4 and tetradecylamine (51%). (MALDI, positive mode, matrix DHB): *m*/*z* = 446 (M + Na)^+^.

### 3-(4-Chlorophenyl)-*N*-cyclohexyl-3-hydroxy-2,2-dimethylpropanamide (7e)

White crystals (81%), mp: 105–107 °C. ^1^H NMR spectrum, (400 MHz, CDCl_3_), *δ*, ppm (*J*, Hz): 7.27–7.21 (5H, m, ArH, NH), 5.73 (1H, bs, OH), 4.59 (1H, s, CH), 3.77–3.75 (1H, m, CH), 1.85–1.81 (4H, m, 2CH_2_), 1.67–1.61 (4H, m, 2CH_2_), 1.38–1.32 (2H, m, CH_2_), 1.22 (3H, s, CH_3_), 1.03 (3H, s, CH_3_). ^13^C NMR spectrum, (100.0 MHz, CDCl_3_), *δ*, ppm: 176.3 (C=O), 138.8, 134.1, 129.1, 128.8, 126.2, 126.0 (C-Ar), 79.1 (CH), 60.3 (C), 51.2 (CH), 33.2 (CH_2_), 33.0 (CH_2_), 25.3 (CH_2_), 24.1 (CH_2_), 23.2 (CH_2_), 22.4 (CH_3_), 19.8 (CH_3_). (MALDI, positive mode, matrix DHB): *m*/*z* = 332 (M + Na)^+^.

Method B, from 6 and cyclohexylamine (72%).

### *N*-Benzyl-3-(4-chlorophenyl)-3-hydroxy-2,2-dimethylpropanamide (7f)

White crystals (83%), mp: 125–126 °C. ^1^H NMR spectrum, (400 MHz, CDCl_3_), *δ*, ppm (*J*, Hz): 7.28–7.10 (9H, m, ArH), 6.08 (1H, bs, NH), 4.60 (1H, m, CH), 4.37 (1H, d, *J* = 8.0 Hz, CH_2_Ph), 4.33 (1H, d, *J* = 8.0 Hz, CH_2_Ph), 3.09 (1H, bs, OH), 1.18 (3H, s, CH_3_), 1.02 (3H, s, CH_3_). ^13^C NMR spectrum, (100.0 MHz, CDCl_3_), *δ*, ppm: 177.3 (C=O), 139.3, 137.8, 133.5, 132.4, 131.7, 129.0, 128.8, 128.3 (2C), 128.0 (2C), 127.7 (C-Ar), 79.4 (CH), 60.3 (C), 43.6 (CH_2_), 22.2 (CH_3_), 20.7 (CH_3_). (MALDI, positive mode, matrix DHB): *m*/*z* = 340 (M + Na)^+^.

Method B, from 6 and benzylamine (76%).

### 3-(4-Chlorophenyl)-3-hydroxy-2,2-dimethyl-1-(piperidin-1-yl)propan-1-one (7g)

White crystals (68%), mp: 162–164 °C. ^1^H NMR spectrum, (400 MHz, CDCl_3_), *δ*, ppm (*J*, Hz): 7.46–7.18 (4H, m, ArH), 5.63 (1H, bs, OH), 4.52–4.50 (1H, m, CH), 3.51–3.47 (4H, m, 2NCH_2_), 1.58–1.47 (6H, m, 3CH_2_), 1.09 (3H, s, CH_3_), 0.89 (3H, s, CH_3_). ^13^C NMR spectrum, (100.0 MHz, CDCl_3_), *δ*, ppm: 178.3 (C=O), 1375, 133.2, 130.0, 129.5, 128.4, 127.9 (C-Ar), 79.4 (CH), 48.2 (NCH_2_), 48.0 (NCH_2_), 45.3 (C), 30.3 (CH_2_), 29.7 (2CH_2_), 20.7 (CH_3_), 20.0 (CH_3_). (MALDI, positive mode, matrix DHB): *m*/*z* = 318 (M + Na)^+^.

Method B, from 6 and piperidin (52%).

### 3-(4-Chlorophenyl)-3-hydroxy-2,2-dimethyl-1-morpholinopropan-1-one (7h)

White crystals (62%), mp: 134–135 °C. ^1^H NMR spectrum, (400 MHz, CDCl_3_), *δ*, ppm (*J*, Hz): 7.22–7.13 (4H, m, ArH), 5.85 (1H, bs, OH), 4.63–4.60 (1H, m, CH), 3.85–3.59 (4H, m, 2CH_2_), 3.37–3.29 (4H, m, 2CH_2_), 1.10 (3H, s, CH_3_), 0.95 (3H, s, CH_3_). ^13^C NMR spectrum, (100.0 MHz, CDCl_3_), *δ*, ppm: 175.6 (C=O), 138.7, 132.1, 129.0, 128.5, 127.1, 126.8 (C-Ar), 80.2 (CH), 65.7 (OCH_2_), 65.5 (OCH_2_), 47.7 (NCH_2_), 45.3 (NCH_2_), 44.8 (C), 21.3 (CH_3_), 19.9 (CH_3_). (MALDI, positive mode, matrix DHB): *m*/*z* = 320 (M + Na)^+^.

Method B, from 6 and morpholine (69%).

### 3-(4-Chlorophenyl)-3-hydroxy-2,2-dimethyl-1-(4-methylpiperazin-1-yl)propan-1-one (7i)

White crystals (67%), mp: 156–157 °C. ^1^H NMR spectrum, (400 MHz, CDCl_3_), *δ*, ppm (*J*, Hz): 7.41–7.35 (4H, m, ArH), 5.83 (1H, bs, OH), 4.71 (1H, s, CH), 3.31–3.29 (4H, m, 2NCH_2_), 2.35–3.28 (4H, m, 2CH_2_), 2.16 (3H, s, CH_3_), 1.09 (3H, s, CH_3_), 0.89 (3H, s, CH_3_). ^13^C NMR spectrum, (100.0 MHz, CDCl_3_), *δ*, ppm: 176.7 (C=O), 140.2, 135.4, 132.8, 128.9, 127.8, 127.2, (C-Ar), 79.2 (CH), 50.5 (NCH_2_), 50.1 (NCH_2_), 49.3 (CH_2_), 49.1 (NCH_2_), 42.4 (C), 41.2 (NCH_3_), 21.1 (CH_3_), 20.2 (CH_3_). (MALDI, positive mode, matrix DHB): *m*/*z* = 333 (M + Na)^+^.

Method B, from 6 and methylpiperazin (58%).

### Preparation of methyl-2-[(3-(4-chlorophenyl)-3-hydroxy-2,2-dimethylpropanoyl)-amino]alkanoates 8a–g

To the previously prepared cold ethyl acetate solution of azide 5 (0.24 g, 1.0 mmol hydrazide), a solution of amino acid esters (1.2 mmol) in ethyl acetate (10 mL) was added dropwise with stirring. The reaction mixture was kept at −5 °C for 12 h, then at room temperature for another 12 h. The reaction mixture was washed with 0.5 N HCl, water and 3% solution of NaHCO_3_ and dried over Na_2_SO_4_. The solution was evaporated to dryness, and the residue was recrystallized from ethyl acetate/petroleum ether to give the desired product 8a–g.

### Methyl-2-[(3-(4-chlorophenyl)-3-hydroxy-2,2-dimethylpropanoyl)amino]acetate (8a)

White crystals (72%), mp: 168–170 °C. ^1^H NMR spectrum, (400 MHz, CDCl_3_), *δ*, ppm (*J*, Hz): 7.32–7.15 (4H, m, ArH), 6.67 (1H, t, *J* = 4.0 Hz, NH), 5.22 (1H, bs, OH), 4.63 (1H, s, CH), 3.92 (2H, t, *J* = 4.0 Hz, NCH_2_), 3.68 (3H, s, OCH_3_), 1.08 (3H, s, CH_3_), 0.81 (3H, m, CH_3_). ^13^C NMR spectrum, (100.0 MHz, CDCl_3_), *δ*, ppm: 177.6 (C=O), 170.1 (C=O), 139.1, 133.2, 129.0, 128.5, 127.6, 127.4 (C-Ar), 79.7 (CH), 51.5 (OCH_3_), 45.2 (C), 41.4 (NCH_2_), 20.1 (CH_3_), 19.8 (CH_3_). (MALDI, positive mode, matrix DHB): *m*/*z* = 322 (M + Na)^+^.

Method B, from 6 and glycine methyl ester hydrochloride (66%).

### Methyl-3-[(3-(4-chlorophenyl)-3-hydroxy-2,2-dimethylpropanoyl)amino]propanoate (8b)

White crystals (86%), mp: 139–141 °C. ^1^H NMR spectrum, (400 MHz, CDCl_3_), *δ*, ppm (*J*, Hz): 7.10–6.88 (4H, m, ArH), 6.87 (1H, bs, NH), 4.76 (1H, bs, OH), 4.47 (1H, s, CH), 3.48 (3H, s, OCH_3_), 3.26 (2H, t, *J* = 4.0 Hz, NCH_2_), 2.33 (2H, t, *J* = 4.0 Hz, CH_2_), 1.19 (3H, s, CH_3_), 0.87 (3H, m, CH_3_). ^13^C NMR spectrum, (100.0 MHz, CDCl_3_), *δ*, ppm: 181.0 (C=O), 171.2 (C=O), 139.6, 132.9, 129.5, 128.8, 128.3, 127.6 (C-Ar), 78.3 (CH), 53.6 (OCH_3_), 44.5 (C), 42.4 (NCH_2_), 33.4 (CH_2_), 20.3 (CH_3_), 20.0 (CH_3_). (MALDI, positive mode, matrix DHB): *m*/*z* = 336 (M + Na)^+^.

Method B, from 6 and β-alanine methyl ester hydrochloride (74%).

### Methyl-4-[(3-(4-chlorophenyl)-3-hydroxy-2,2-dimethylpropanoyl)amino]butanoate (8c)

White crystals (74%), mp: 139–141 °C. ^1^H NMR spectrum, (400 MHz, CDCl_3_), *δ*, ppm (*J*, Hz): 7.42–7.21 (4H, m, ArH), 6.63 (1H, bs, NH), 4.82 (1H, bs, OH), 4.61 (1H, s, CH), 3.56 (3H, s, OCH_3_), 3.20 (2H, m, NCH_2_), 2.36 (2H, m, CH_2_), 2.11 (2H, m, CH_2_), 1.21 (3H, s, CH_3_), 0.91 (3H, m, CH_3_). ^13^C NMR spectrum, (100.0 MHz, CDCl_3_), *δ*, ppm: 179.3 (C=O), 174.7 (C=O), 138.4, 133.6, 130.5, 129.2, 128.8, 128.0 (C-Ar), 79.6 (CH), 55.2 (OCH_3_), 45.8 (C), 41.2 (NCH_2_), 30.0 (CH_2_), 24.2 (CH_2_), 20.0 (CH_3_), 19.7 (CH_3_). (MALDI, positive mode, matrix DHB): *m*/*z* = 350 (M + Na)^+^.

Method B, from 6 and γ-amino butyric methyl ester hydrochloride (61%).

### Methyl-2-[(3-(4-chlorophenyl)-3-hydroxy-2,2-dimethylpropanoyl)amino]propanoate (8d)

White crystals (69%), mp: 88–90 °C. ^1^H NMR spectrum, (400 MHz, CDCl_3_), *δ*, ppm (*J*, Hz): 7.42–7.21 (4H, m, ArH), 6.54 (1H, bs, NH), 5.53 (1H, bs, OH), 4.54 (1H, s, CH), 4.41 (1H, m, NCH), 3.58 (3H, s, OCH_3_), 1.34 (3H, s, CH_3_), 1.17 (3H, s, CH_3_), 0.99 (3H, m, CH_3_). ^13^C NMR spectrum, (100.0 MHz, CDCl_3_), *δ*, ppm: 176.8 (C=O), 172.7 (C=O), 138.4, 134.0, 130.4, 129.5, 128.7, 127.2 (C-Ar), 80.2 (CH), 54.8 (OCH_3_), 48.3 (NCH), 45.9 (C), 20.0 (CH_3_), 19.6 (CH_3_), 18.5 (CH_3_). (MALDI, positive mode, matrix DHB): *m*/*z* = 336 (M + Na)^+^.

Method B, from 6 and l-alanine methyl ester hydrochloride (57%).

### Methyl-2-[(3-(4-chlorophenyl)-3-hydroxy-2,2-dimethylpropanoyl)amino]3-methyl butanoate (8e)

White crystals (57%), mp: 109–111 °C. ^1^H NMR spectrum, (400 MHz, CDCl_3_), *δ*, ppm (*J*, Hz): 7.35–7.19 (4H, m, ArH), 6.86 (1H, bs, NH), 5.27 (1H, bs, OH), 4.87 (1H, s, CH), 4.52–4.45 (1H, m, NCH), 3.71 (3H, s, OCH_3_), 2.16–2.13 (1H, m, CH), 1.10 (3H, s, CH_3_), 0.98–0.86 (9H, m, 3CH_3_). ^13^C NMR spectrum, (100.0 MHz, CDCl_3_), *δ*, ppm: 176.8 (C=O), 173.2 (C=O), 139.5, 133.8, 129.2, 128.9, 126.7, 127.4 (C-Ar), 79.7 (CH), 55.1 (OCH_3_), 51.2 (NCH), 46.2 (C), 31.2 (CH), 20.2 (CH_3_), 19.7 (CH_3_), 18.6 (CH_3_), 18.4 (CH_3_). (MALDI, positive mode, matrix DHB): *m*/*z* = 364 (M + Na)^+^.

Method B, from 6 and l-valine methyl ester hydrochloride (55%).

### Methyl-2-[(3-(4-chlorophenyl)-3-hydroxy-2,2-dimethylpropanoyl)amino]4-methyl pentanoate (8f)

White crystals (64%), mp: 68–70 °C. ^1^H NMR spectrum, (400 MHz, CDCl_3_), *δ*, ppm (*J*, Hz): 7.35–7.19 (4H, m, ArH), 6.86 (1H, bs, NH), 5.27 (1H, bs, OH), 4.87 (1H, s, CH), 4.52–4.45 (1H, m, NCH), 3.71 (3H, s, OCH_3_), 2.16–2.13 (1H, m, CH), 1.97–1.93 (2H, m, CH_2_), 1.10 (3H, s, CH_3_), 0.98–0.86 (9H, m, 3CH_3_). ^13^C NMR spectrum, (100.0 MHz, CDCl_3_), *δ*, ppm: 176.8 (C=O), 173.2 (C=O), 139.5, 133.8, 129.2, 128.9, 126.7, 127.4 (C-Ar), 79.7 (CH), 55.1 (OCH_3_), 51.2 (NCH), 46.2 (C), 38.3 (CH_2_), 31.2 (CH), 20.2 (CH_3_), 19.7 (CH_3_), 18.6 (CH_3_), 18.4 (CH_3_). (MALDI, positive mode, matrix DHB): *m*/*z* = 378 (M + Na)^+^.

Method B, from 6 and l-leucine methyl ester hydrochloride (53%).

### Methyl-2-[(3-(4-chlorophenyl)-3-hydroxy-2,2-dimethylpropanoyl)amino]4-methyl-sulfanylbutanoate (8g)

White crystals (59%), mp: 96–98 °C. ^1^H NMR spectrum, (400 MHz, CDCl_3_), *δ*, ppm (*J*, Hz): 7.38–7.15 (4H, m, ArH), 6.73 (1H, bs, NH), 5.35 (1H, bs, OH), 4.76 (1H, s, CH), 4.63–4.57 (1H, m, NCH), 3.61 (3H, s, OCH_3_), 2.58–2.51 (2H, m, CH_2_), 2.19–2.14 (2H, m, CH_2_), 2.10 (3H, s, CH_3_), 1.14 (3H, s, CH_3_), 0.87 (3H, m, CH_3_). ^13^C NMR spectrum, (100.0 MHz, CDCl_3_), *δ*, ppm: 176.8 (C=O), 173.2 (C=O), 139.5, 133.8, 129.2, 128.9, 126.7, 127.4 (C-Ar), 79.7 (CH), 55.1 (OCH_3_), 51.2 (NCH), 46.2 (C), 33.7 (CH_2_), 32.3 (CH_2_), 20.6 (CH_3_), 19.3 (CH_3_), 18.8 (CH_3_). (MALDI, positive mode, matrix DHB): *m*/*z* = 396 (M + Na)^+^.

Method B, from 6 and l-methionine methyl ester hydrochloride (48%).

### Preparation of methyl-3-(4-chlorophenyl)-2,2-dimethyl-3-(2,2,2-trichloro-1-iminoethoxy)propanoate (9)

A stirred solution of methyl 3-(4-chlorophenyl)-3-hydroxy-2,2-dimethylpropanoate (3) (1.0 mmol) in dry dichloromethane (30 mL) was treated with trichloroacetonitrile (0.3 mL, 2.0 mmol) and DBU (0.1 mL, 0.5 mmol). The reaction mixture was stirred at room temperature for 2 h. The solvent was evaporated under reduced pressure and the product was purified by column chromatography 5% triethylamine in dichloromethane, to give 9 as whitish oil (82%). ^1^H NMR spectrum, (400 MHz, CDCl_3_), *δ*, ppm (*J*, Hz): 8.65 (1H, bs, NH), 7.47–7.18 (4H, m, ArH), 5.21 (1H, s, CH), 3.73 (3H, s, OCH_3_), 1.11 (3H, s, CH_3_), 0.90 (3H, m, CH_3_). ^13^C NMR spectrum, (100.0 MHz, CDCl_3_), *δ*, ppm: 177.3 (C=O), 168.7 (C=NH), 134.8, 132.5, 129.1, 128.4, 128.0, 127.7 (C-Ar), 88.7 (CCl_3_), 79.0 (CH), 53.8 (OCH_3_), 45.9 (C), 20.0 (CH_3_), 19.6 (CH_3_). (MALDI, positive mode, matrix DHB): *m*/*z* = 408 (M + Na)^+^.

### Preparation of methyl 3-acetoxy-3-(4-chlorophenyl)-2,2-dimethylpropanoate (10)

To a solution of methyl 3-(4-chlorophenyl)-3-hydroxy-2,2-dimethylpropanoate (3) (0.24 g, 1.0 mmol) in dichloromethane, acetic anhydride (1.2 mmol) and dimethyl aminopyridine (DMAP) (0.2 mmol) was added. The reaction mixture was stirred at room temperature for 4 h and was evaporated under reduced pressure to give oily residue. The residue was purified by column chromatography 4 : 1 pet. ether/ethyl-acetate, to give 10 as white powder (82%).

White crystals (89%), mp: 61–63 °C. ^1^H NMR spectrum, (400 MHz, CDCl_3_), *δ*, ppm (*J*, Hz): 7.28–7.19 (4H, m, ArH), 6.01 (1H, s, CH), 3.66 (3H, s, OCH_3_), 2.05 (3H, s, CH_3_), 1.22 (3H, s, CH_3_), 1.09 (3H, s, CH_3_). ^13^C NMR spectrum, (100.0 MHz, CDCl_3_), *δ*, ppm: 176.4 (C=O), 169.3 (C=O), 135.5, 133.9, 131.4, 128.6, 128.1, 127.4 (C-Ar), 78.4 (CH), 51.9 (OCH_3_), 47.0 (C), 21.9 (CH_3_), 21.7 (CH_3_), 20.1 (CH_3_). (MALDI, positive mode, matrix DHB): *m*/*z* = 307 (M + Na)^+^.

### Preparation of methyl-3-aryl-3-(4-chlorophenyl)-2,2-dimethylpropanoate 11a–e

A solution of trichloroacetimidate 9 (method A) or acetate 10 (method B) (1.4 mmol) in dichloromethane (10 mL) was added C-nucleophiles (1.4 mmol); anisole, 1,4-dimethoxybenzene, 1,2-dimethoxybenzene and 1,2,3-trimethoxybenzene and TMSOTf (13 μL, 0.06 mmol). The reaction mixture was stirred at room temperature under dry nitrogen till completion of the reaction (TLC monitored and the time was recorded). The reaction mixture was neutralized with solid sodium bicarbonate, filtered and concentrated in vacuum. The residue was purified by column chromatography 3 : 1 pet. ether/ethyl-acetate as eluent, to give 11a–e.

### Methyl-3-(4-chlorophenyl)-3-(4-methoxyphenyl)-2,2-dimethylpropanoate (11a)

Oil (84%). ^1^H NMR spectrum, (400 MHz, CDCl_3_), *δ*, ppm (*J*, Hz): 7.21–7.08 (6H, m, ArH), 7.06–6.99 (2H, m, ArH), 4.23 (1H, s, CH), 3.68 (3H, s, OCH_3_), 3.44 (3H, s, OCH_3_), 1.16 (3H, s, CH_3_), 1.02 (3H, s, CH_3_). ^13^C NMR spectrum, (100.0 MHz, CDCl_3_), *δ*, ppm: 176.2 (C=O), 146.8, 140.3, 135.6, 132.5, 129.4, 129.0, 128.1, 127.8, 125.5, 125.2, 127.1, 116.9 (C-Ar), 56.1 (OCH_3_), 53.2 (OCH_3_), 48.5 (CH), 47.2 (C), 20.2 (CH_3_), 19.6 (CH_3_). (MALDI, positive mode, matrix DHB): *m*/*z* = 355 (M + Na)^+^.

Method B, oil (77%).

### Methyl-3-(4-chlorophenyl)-3-(2,5-dimethoxyphenyl)-2,2-dimethylpropanoate (11b)

Oil (78%). ^1^H NMR spectrum, (400 MHz, CDCl_3_), *δ*, ppm (*J*, Hz): 7.21–7.10 (4H, m, ArH), 6.75–6.66 (3H, m, ArH), 4.43 (1H, s, CH), 3.65 (3H, s, OCH_3_), 3.63 (3H, s, OCH_3_), 3.59 (3H, s, OCH_3_), 1.13 (3H, s, CH_3_), 1.01 (3H, s, CH_3_). ^13^C NMR spectrum, (100.0 MHz, CDCl_3_), *δ*, ppm: 176.5 (C=O), 148.2, 146.4, 145.2, 145.0, 141.3, 139.5, 133.4, 133.1, 129.2, 126.5, 118.7, 116.2 (C-Ar), 56.2 (OCH_3_), 55.4 (OCH_3_), 52.8 (OCH_3_), 49.8 (C), 44.2 (CH), 21.1 (CH_3_), 20.3 (CH_3_). (MALDI, positive mode, matrix DHB): *m*/*z* = 385 (M + Na)^+^.

Method B, oil (65%)

### Methyl-3-(4-chlorophenyl)-3-(2,3-dimethoxyphenyl)-2,2-dimethylpropanoate (11c)

Oil (81%). ^1^H NMR spectrum, (400 MHz, CDCl_3_), *δ*, ppm (*J*, Hz): 7.23–7.13 (4H, m, ArH), 6.81–6.80 (3H, m, ArH), 4.51 (1H, s, CH), 3.68 (3H, s, OCH_3_), 3.64 (6H, s, 2OCH_3_), 1.22 (3H, s, CH_3_), 1.03 (3H, s, CH_3_). ^13^C NMR spectrum, (100.0 MHz, CDCl_3_), *δ*, ppm: 176.4 (C=O), 149.3, 147.8, 140.2, 140.0, 139.5, 135.7, 131.0, 129.8, 129.1, 123.2, 119.4, 116.0 (C-Ar), 57.7 (OCH_3_), 56.5 (OCH_3_), 54.2 (OCH_3_), 47.9 (C), 45.3 (CH), 20.8 (CH_3_), 19.9 (CH_3_). (MALDI, positive mode, matrix DHB): *m*/*z* = 385 (M + Na)^+^.

Method B, oil (76%)

### Methyl-3-(4-chlorophenyl)-3-(2,4-dimethoxyphenyl)-2,2-dimethylpropanoate (11d)

Oil (80%). ^1^H NMR spectrum, (400 MHz, CDCl_3_), *δ*, ppm (*J*, Hz): 7.39–7.22 (4H, m, ArH), 7.09–6.79 (3H, m, ArH), 4.53 (1H, s, CH), 3.81 (6H, s, 2OCH_3_), 3.61 (3H, s, OCH_3_), 1.20 (3H, s, CH_3_), 1.08 (3H, s, CH_3_). ^13^C NMR spectrum, (100.0 MHz, CDCl_3_), *δ*, ppm: 174.1 (C=O), 153.9, 150.6, 149.3, 138.2, 133.5, 132.1, 130.3, 129.1, 127.5, 119.7, 114.6, 112.0 (C-Ar), 58.2 (OCH_3_), 57.2 (OCH_3_), 55.7 (OCH_3_), 48.2 (C), 47.5 (CH), 21.7 (CH_3_), 19.2 (CH_3_). (MALDI, positive mode, matrix DHB): *m*/*z* = 385 (M + Na)^+^.

Method B, oil (69%)

### Methyl-3-(4-chlorophenyl)-2,2-dimethyl-3-(3,4,5-trimethoxyphenyl)propanoate (11e)

Oil (88%). ^1^H NMR spectrum, (400 MHz, CDCl_3_), *δ*, ppm (*J*, Hz): 7.30–7.20 (4H, m, ArH), 7.00–6.60 (2H, m, ArH), 4.62 (1H, s, CH), 3.88 (9H, s, 3OCH_3_), 3.68 (3H, s, OCH_3_), 1.22 (3H, s, CH_3_), 1.10 (3H, s, CH_3_). ^13^C NMR spectrum, (100.0 MHz, CDCl_3_), *δ*, ppm: 176.1 (C=O), 152.4, 151.9, 150.0, 136.0, 131.8, 130.0, 129.9, 129.7, 128.1, 127.3, 113.8, 112.1 (C-Ar), 58.5 (OCH_3_), 57.2 (OCH_3_), 56.1 (OCH_3_), 54.5 (OCH_3_), 48.4 (C), 47.6 (CH), 21.3 (CH_3_), 20.5 (CH_3_). (MALDI, positive mode, matrix DHB): *m*/*z* = 415 (M + Na)^+^.

Method B, oil (74%).

## Experimental method

### Antiproliferative activity by MTT assay

The antiproliferative activity of both non-cancerous cells (human embryonic kidney cells – HEK-293) and cancerous cells (human colorectal carcinoma cells – HCT-116) was done by MTT assay. The cell lines (human embryonic kidney cells – HEK-293) and cancerous cells (human colorectal carcinoma cells – HCT-116) used in the study were obtained from Dr Khaldoon M. Alsamman, Clinical Laboratory Science, College of Applied Medical Science, Imam Abdulrahman Bin Faisal University, Dammam, Saudi Arabia. The cell culture and MTT assay was done as per previously described method.^39^ However, we have described the method in brief. Cells were grown in 96-well plates in the culture media containing DMEM, l-glutamine, fetal bovine serum, selenium chloride and penicillin–streptomycin. The cells were treated with different concentrations (0.06 mg mL^−1^ to 0.7 mg mL^−1^) of 15 synthetic compounds. In the control group, no synthetic compounds were added. After 48 hours of treatments, MTT (5.0 mg mL^−1^) was added in each well and were kept incubated for 4 hours. Thereafter, DMSO was added and plates were read at 570 nm wavelength using ELISA Plate Reader (Biotek Instruments, Winooski, USA). The percentage (%) of cell viability (%) was calculated and all statistical analyses were completed with GraphPad Prism 6 (GraphPad Software). The difference between control and compound-treated groups by a one-way analysis of variance (ANOVA), and *p*-values were calculated by Student's *t*-test.

### Nuclear staining

We examined status of nuclear disintegration of cancerous cells after treatments by staining with DAPI (4′,6-diamidino-2-phenylindole). We have selected compounds 2P and 55P which showed highest inhibitory action on the HCT-116 cell for the DAPI staining. The cancerous (HCT-116) cells were treated with (0.1 mg mL^−1^) concentration. In control group, compounds 2P and 55P were not added. After 48 hours of the treatment, both control and compounds 2P and 55P-treated groups were immersed in 4% paraformaldehyde solution. Thereafter, fixed cells were treated with Triton X-100 + phosphate buffered saline (PBS). Both control and compounds 2P and 55P-treated cells were stained with DAPI. The nuclear staining of both control and MNPs-treated cells was examined under confocal scanning microscope (Zeiss Germany).

### Molecular modeling

All molecular modeling studies were performed on a Hewlett-Packard Pentium Dual-Core T4300 2.10 GHz running Windows 10 Ultimate using autodock 4.3 for molecular docking simulation and ligand binding energy calculation. The crystal structure of HSP90 (PDB code; 2xab) and TRAP1 (PDB code; 5y3n) co-crystallized with inhibitor was used as receptor in the docking studies. The selected target was used after deleting the co-crystallized inhibitor. Docking calculations were carried out using the AutoDock 4.3 software (La Jolla, CA). First all hydrogens were added to the ligand PDB file and Gasteiger charges were computed and all the torsion angles of the ligand were defined with the autodock-tools program so they could be explored during molecular modeling. A grid box of 51.3260 × 45.8986 × 25.0000 Å and 88.6644 × 111.8783 × 25.0000 Å for HSP90 and TRAP1 respectively to incorporate the whole protein to further evaluate the selectivity of the tested compounds was used to calculate the atom types needed for the calculation. The Lamarckian genetic algorithm was used as a search method with a total of 30 runs (maximum of 20 000 000 energy evaluations; 27 000 generations; initial populations of 150 conformers). The docking results were evaluated using binding energy calculation in autodock and checking ligand binding position visually in Pymol.

## Conclusion

A series of 24 compounds were synthesized based on structure modification of the model methyl-3-(4-chlorophenyl)-3-hydroxy-2,2-dimethylpropanoate as potent HDACIs. Methyl-3-(4-chlorophenyl)-3-hydroxy-2,2-dimethylpropanoate was transformed into *N*-alkyl-3-(4-chlorophenyl)-3-hydroxy-2,2-dimethylpropanamides and methyl-2-[(3-(4-chlorophenyl)-3-hydroxy-2,2-dimethylpropanoyl)amino]alkanoates *via* DCC and azide by the reaction with amines and amino acid esters, respectively. Methyl-3-(4-chlorophenyl)-3-hydroxy-2,2-dimethylpropanoate reacted with acetic anhydride or trichloroacetonitrile to afford acetate or trichloroacetimidate, which was further reacted with C-active nucleophiles in the presence of TMSOTf (0.1 eq.%) to afford methyl-3-aryl-3-(4-chlorophenyl)-2,2-dimethylpropanoates *via* C–C bond formation. With careful design and optimization of those compounds especially those with the amino acid side chain we would be able to develop a potential candidate that has enough selectivity to progress to clinical trials. Optimization of compounds 7a and 7g for enhanced TRAP1 activity represents our future research direction. The 48 post-treatments showed that out of 24 compounds, 12 compounds showed inhibitory actions on HCT-116 cells, we have calculated the inhibitory action (IC_50_) of these compounds on both HCT-116 and we have found that IC_50_ were in between is 0.12 mg mL^−1^ to 0.81 mg mL^−1^. The compounds (7a & 7g) showed highest inhibitory activity (0.12 mg mL^−1^), whereas compound 7d showed lowest inhibitory activity (0.81 mg mL^−1^). The treated cancerous cells were also examined for nuclear disintegration through staining with DAPI, and we have found that there was loss of DAPI staining in the treated cancerous cells. We have also examined inhibitory action on normal and non-cancerous cells (HEK-293 cells) and confirmed that action of these compounds was specific to cancerous cells.

## Conflicts of interest

There are no conflicts to declare.

## Supplementary Material
